# Beyond autophagy: a novel role for autism-linked Wdfy3 in brain mitophagy

**DOI:** 10.1038/s41598-018-29421-7

**Published:** 2018-07-27

**Authors:** Eleonora Napoli, Gyu Song, Alexios Panoutsopoulos, M. Asrafuzzaman Riyadh, Gaurav Kaushik, Julian Halmai, Richard Levenson, Konstantinos S. Zarbalis, Cecilia Giulivi

**Affiliations:** 1 Department of Molecular Biosciences, School of Veterinary Medicine, University of California, Davis, CA 95616 USA; 2 Department of Pathology and Laboratory Medicine, University of California Davis, Davis, USA; 3 Institute for Pediatric Regenerative Medicine, Shriners Hospitals for Children, Northern California, 2425 Stockton Boulevard, Sacramento, CA 95817 USA; 4 Medical Investigations of Neurodevelopmental Disorders (MIND) Institute, University of California Davis, Davis, CA 95817 USA

## Abstract

WD repeat and FYVE domain-containing 3 (*WDFY3*; also known as Autophagy-Linked FYVE or Alfy) is an identified intellectual disability, developmental delay and autism risk gene. This gene encodes for a scaffolding protein that is expressed in both the developing and adult central nervous system and required for autophagy and aggrephagy with yet unexplored roles in mitophagy. Given that mitochondrial trafficking, dynamics and remodeling have key roles in synaptic plasticity, we tested the role of Wdfy3 on brain bioenergetics by using *Wdfy3*^+/*lacZ*^ mice, the only known *Wdfy3* mutant animal model with overt neurodevelopmental anomalies that survive to adulthood. We found that Wdfy3 is required for sustaining brain bioenergetics and morphology via mitophagy. Decreased mitochondrial quality control by conventional mitophagy was partly compensated for by the increased formation of mitochondria-derived vesicles (MDV) targeted to lysosomal degradation (micromitophagy). These observations, extended through proteomic analysis of mitochondria-enriched cortical fractions, showed significant enrichment for pathways associated with mitophagy, mitochondrial transport and axon guidance via semaphorin, Robo, L1cam and Eph-ephrin signaling. Collectively, our findings support a critical role for Wdfy3 in mitochondrial homeostasis with implications for neuron differentiation, neurodevelopment and age-dependent neurodegeneration.

## Introduction

The human central nervous system has relatively high energy demands, with approximately 20% of total metabolic expenditure being incurred by about 2% of body mass. The majority of this energy is spent on the principal neuronal function of firing action potentials and neuronal communication through chemical synapses by providing support to the Na^+^, K^+^-ATPase^1^. Mitochondria, via the generation of adenosine triphosphate (ATP) through a process named oxidative phosphorylation (OXPHOS), sustains the Na^+^, K^+^-ATPase activity and cellular growth and proliferation. Based on the critical role of mitochondria in neurons, mitochondrial dysfunction has been associated with several neurological and neurodevelopmental disorders as well as major psychiatric illnesses, including depression^2^, schizophrenia^3^ and autism spectrum disorder (ASD)^4^. Adequate mitochondrial function relies on the fine balance between mitochondrial biogenesis and the selective autophagic clearance of damaged mitochondria or mitophagy^5^. Remarkably, these processes not only preside mitochondrial morphology and number *per* cell, but also cellular bioenergetics. It is therefore not unreasonable to hypothesize that deficits in mitophagy could be associated with impaired cellular energetic balance^6^ and importantly, with clinically significant psychiatric and neurological disorders^7^.

**Figure 1:**
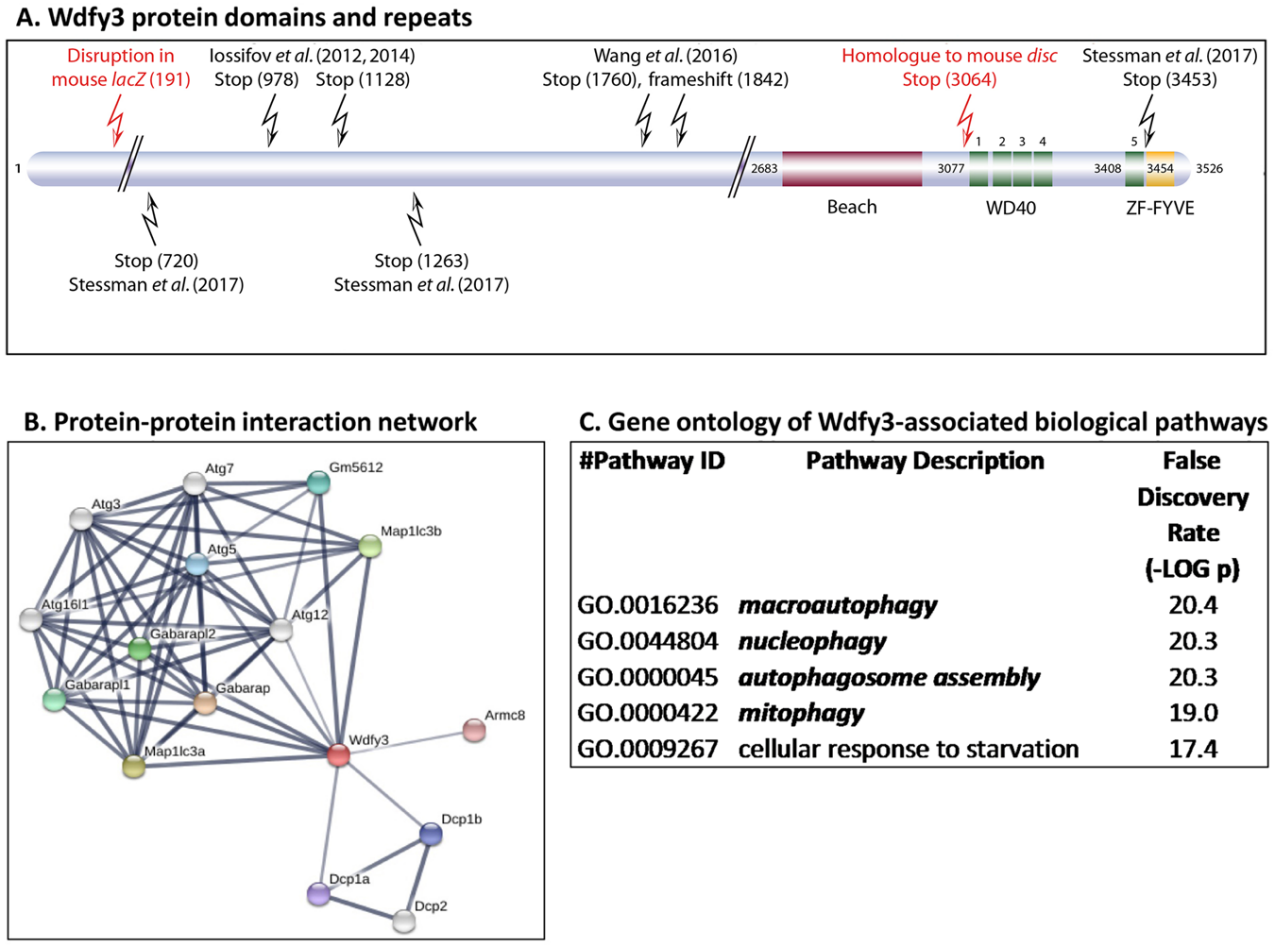
Wdfy3 protein domains, protein network and pathways. (**A**) Schematic representation of human Wdfy3 protein domains and repeats constructed according to InterPro database information. The positions of the pathogenic mutations reported in subjects are indicated on the diagram as well as the homologous positions of the mouse alleles. (**B**) Network of proteins associated with Wdfy3. Network nodes represent proteins whereas the edges represent protein-protein interactions (physical, functional). Analysis was performed with STRING^75^, utilizing the following settings: minimum interaction score of 0.4; first shell of <10 interactors and the second shell with <5. The active interaction sources were text mining, experimental, databases, co-expression, neighborhood, gene fusion and co-occurrence. The line thickness indicates the strength of the data supporting the network edges. (**C**) Gene ontology of biological pathways associated with Wdfy3 from STRING analysis. Pathways were filtered by a false discovery rate <0.05. From these (31 pathways), only the top 5 are shown. In bold and italics, pathways associated with autophagy and mitophagy.

Among the autophagy-regulating factors that play a role in neurodevelopment, brain function and mental health, Wdfy3 (WD repeat and FYVE domain-containing 3, also known as autophagy-linked FYVE or Alfy) is of particular interest. Human *WDFY3* has been shown to be a risk gene for intellectual and developmental disabilities^8^, and has been associated with familial microcephaly^9^ as well as ASD with macrocephaly^10–12^. *Wdfy3* encodes a key adaptor molecule in autophagy likely involved in autophagosome to lysosome fusion^13^. Wdfy3 belongs to the BEACH (**BE**ige **A**nd **CH**S proteins) protein family and contains, in addition to its BEACH domain, five WD40 repeats and a *C*-terminal FYVE (**F**ab1/**Y**OTB/**V**ac1/**E**EA1) domain^14^ (Fig. 1A). Wdfy3 has been shown to interact directly with the lipid membrane component phosphatidylinositol 3-phosphate through its FYVE domain^14^, with Atg5 through its WD repeats (WDR)^13^, and with the ubiquitin-binding protein P62/Sqstm1 via its BEACH domain^15^ (Fig. 1A). In particular, Wdfy3’s interaction with Atg5 connects it with the core macroautophagic machinery through a greater network that involves additional components, including Atg12, Atg16L and LC3 (Fig. 1B). While the exact function of the Wdfy3 BEACH domain is still not completely understood, some insight arises from our previous studies on *Wdfy3*^*disc*^ mice, an animal model that lacks both WD40 repeats and the zinc-finger-FYVE domain (stop at amino acid 3064), but still preserves the BEACH domain resulting in a substantially milder phenotype compared to the *Wdfy3*^*lacZ*^ mice [with disruption at amino acid 191, and hence missing BEACH domain, WD40 repeats and zinc-finger-FYVE domain; Fig. 1A)^16^]. Gene ontology analysis of Wdfy3-associated biological pathways shows significant involvement of Wdfy3 in macroautophagy, but also in selective autophagy (Fig. 1C), a process that involves the elimination of specific cellular structures, which if not properly removed, could lead to cellular damage (e.g., mitophagy, nucleophagy, among others; Fig. 1C).

**Figure 2:**
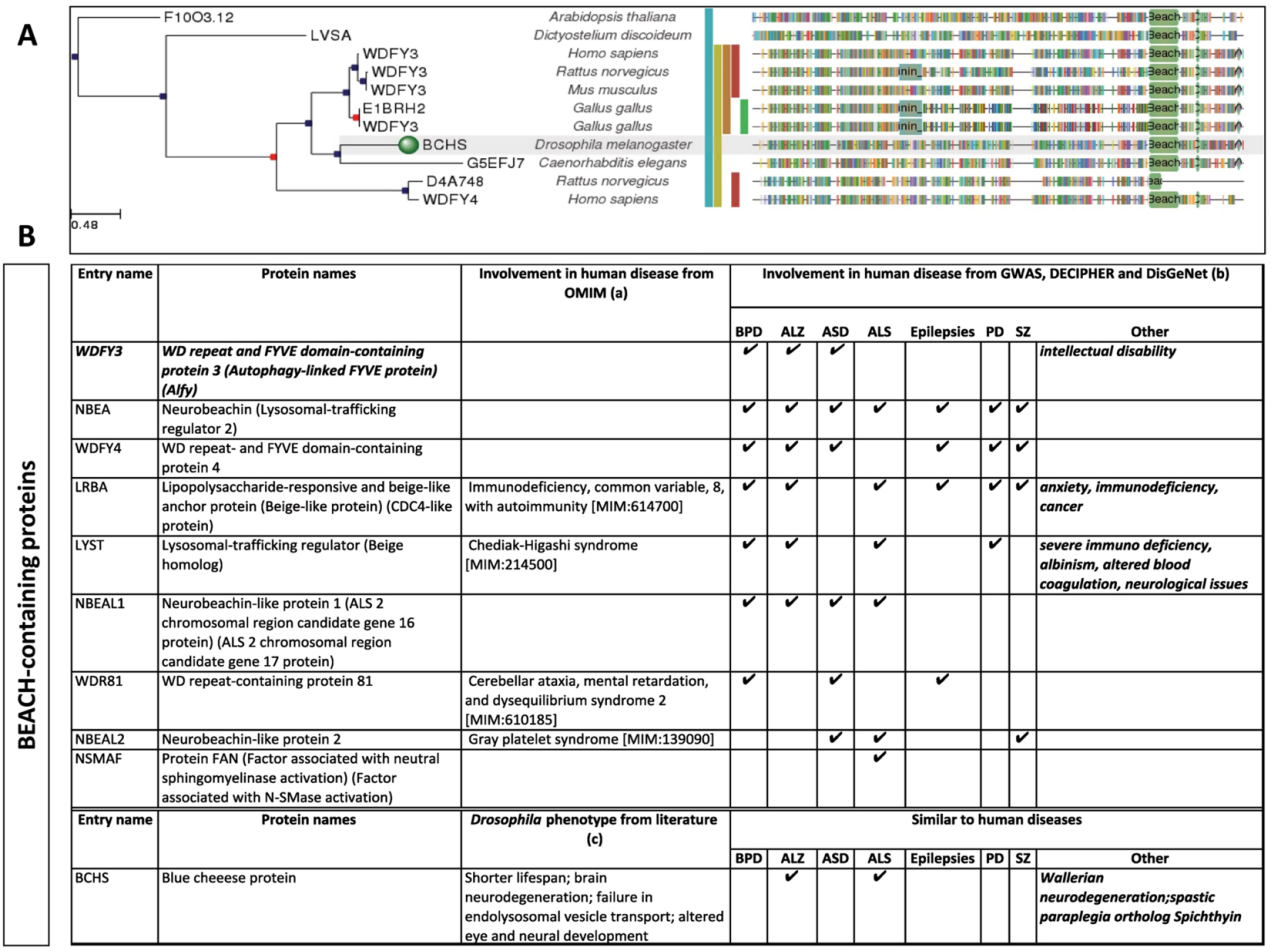
Involvement of BEACH-containing proteins in diseases. (**A**) Homology of human Wdfy3 to other proteins across species (marked with a green circle, the Blue cheese (Bchs) protein in *Drosophila melanogaster*). Analysis obtained with phylomed.org. (**B**) The role of BEACH-containing proteins on human diseases was investigated by performing database mining in (a) OMIM and (b) GWAS, DECIPHER and DIsGeNet. Checkmarks indicate the presence of the disease with that particular protein in any the databases. Abbreviations: bipolar disorder (BPD), Alzheimer’s disease (ALZ); autism spectrum disorders (ASD); amyotrophic lateral sclerosis (ALS); Parkinson’s disease (PD); schizophrenia (SZ). For the case of *Drosophila*, the effect of Bchs deficiency in the context of human disease was obtained from multiple studies^15,19,76,77^.

Recent findings on WDR- and BEACH-containing proteins have shed light on their involvement in neurological diseases. WDR40-47, a microtubule-associated protein, is relevant for both autophagy and brain development^17^, and among the >200 WDR genes annotated in both human and murine genomes, mutations in ~10% of the WDR genes have been implicated in brain disorders, including intellectual disability^17^. Homozygous genetic ablation of *Wdfy3* in mice leads to perinatal lethality and global long-range connectivity defects^18^. Loss of bchs, the Wdfy3 homologous protein in *Drosophila* (Fig. 2A), results in altered endolysosomal transport, neurodegeneration and shorter lifespan^19^, comparable to some human neurodegenerative disorders [e.g., Alzheimer’s disease (AD), amyotrophic lateral sclerosis (ALS), Wallerian neurodegeneration and spastic paraplegia]. Deficits or pathogenic mutations in BEACH-containing proteins other than WDFY3 in humans (Fig. 2B) are associated with bipolar disorder, schizophrenia, epilepsy, ASD, and neurodegenerative diseases, such as AD, ALS and Parkinson’s disease (Fig. 2B).

In the present studies, we investigated the role of Wdfy3 on selective autophagy (mitophagy) by utilizing *Wdfy3*^+/*lacZ*^ mice, the only known heterozygous *Wdfy3* mouse model that displays overt neurodevelopmental anomalies and that also survive to adulthood^16^. The rationale for our search for potential deficits in mitochondrial function of *Wdfy3* haploinsufficiency is based on (i) the high expression of *Wdfy3* in both the developing central nervous system and adult brain^18^; (ii) the critical role of mitochondrial fatty acid β-oxidation at controlling the transition from neural stem cells (NSC) to intermediate progenitor cells (IPC) in the mammalian neocortex^20^; and (iii) the premise that clearance of damaged mitochondria via mitophagy is vital to proper mitochondrial trafficking, an essential aid to brain plasticity^21,22^. To this end, we assessed the role of Wdfy3 in brains from adult *Wdfy3*^+/*lacZ*^ and wild type (WT) mice via detailed biochemical, microscopic, and proteomic methods. Our analysis confirmed deficiencies in mitochondrial function of *Wdfy3*^+/*lacZ*^ mice resulting from defective mitophagy. Our observations indicate that the clearance of damaged mitochondria in the setting of *Wdfy3* haploinsufficiency may be partly abrogated through the formation of mitochondria-derived vesicles (MDV), subsequently targeted to lysosomes for degradation in a process named micromitophagy. We complemented this work by a global proteomics approach which indicated dysregulated cellular pathways consistent with the brain abnormalities and biochemical changes observed in *Wdfy3*^+/*lacZ*^ mice. Specifically, we observed significant over-representation of pathways regulating not only mitophagy and autophagy, but also mitochondrial transport, axonal transport and remodeling of the axonal cytoskeleton, with the involvement of Semaphorin, Robo, L1cam, and Eph-ephrin signaling.

Herein, we present substantial evidence (*in vivo* and *in vitro*) that Wdfy3 is required for mitophagy as well as for mitochondrial transport, likely leading to accumulation of defective mitochondria and impaired brain bioenergetics. The accumulation of defective mitochondria compromises fatty acid β-oxidation, thereby affecting neuron differentiation and/or NSC-to-IPS transition during neurodevelopment. These findings provide a mechanism for the increased developmental neurogenesis due to an enlarged NSC pool that arises through an expansion of self-renewing divisions of radial glial cells with perinatal gains in cerebral length observed in the *Wdfy3*^+/*lacZ*^ mice^16^, and likely a functional link for this gene to intellectual and developmental disabilities as well as ASD.

## Results

### Wdfy3 deficits result in perinatal mortality

All experiments were conducted on WT controls and age-matched *Wdfy3*-haploinsufficient mice due to the perinatal lethality of the homozygous mutants^16^. The C57BL/6NJ genetic background was chosen because the more widely available C57BL/6J mice carry a deletion encompassing the *Nnt* gene resulting in negligible mitochondrial nicotinamide transhydrogenase activity, with consequent increased background-levels of mitochondrial oxidative stress, affecting bioenergetic capabilities^23^.

*Wdfy3*^*lacZ*^ mice, whose generation was previously described in detail^16^, carry a knockout first, *lacZ* reporter-tagged insertion construct integrated between exons 7 and 8 (disruption at aa 191)^24^. Previously reported Western blot analysis confirmed that the *Wdfy3*^*lacZ*^ allele is a hypomorph with overt loss of the largest ~400-kD isoform, but with the retention of smaller bands (possibly isoforms of smaller MW that cross-reacted with the antibody).

**Figure 3:**
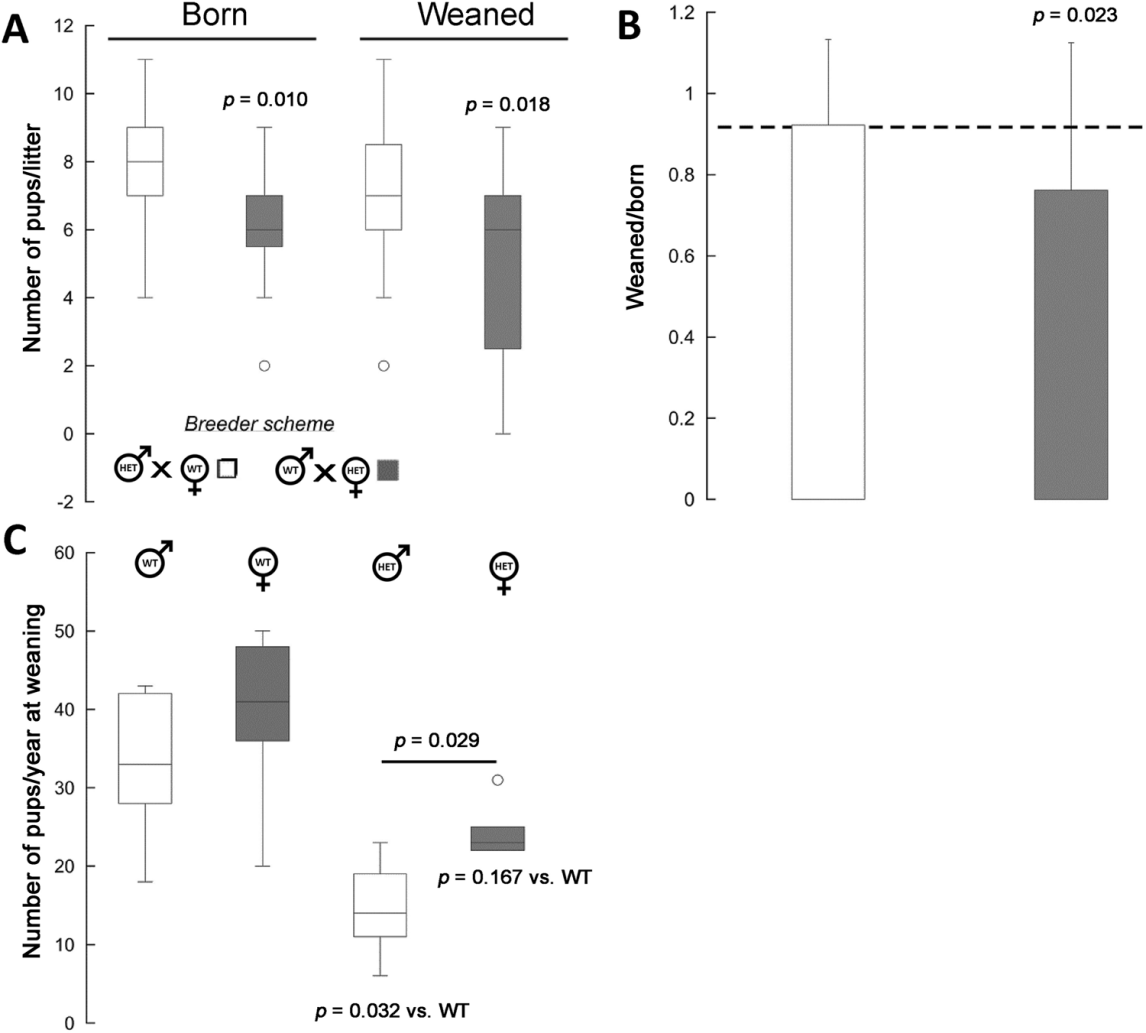
Perinatal mortality and survival of *Wdfy3* insufficient pups. (**A**) Average number of pups born or weaned *per* litter (data collected from 11 WT and 6 *Wdfy3*^*lacZ*^ female breeders, respectively, for each of the breeder schemes) born to either *Wdfy3*^*lacZ*^ male x WT female or *Wdfy3*^*lacZ*^ female x WT male. Later, these pups were utilized for behavioral and biochemical tests. Statistics was performed by Mann-Whitney test. (**B**) Number of live litters on postpartum day 21 (post-weaning) relative to the number of live litters born. Both sets of data were compared to the published value of 0.92 for the C57BL/6J strain. The significant *p* value was obtained by using the test for one proportion (Z-statistic = 2.284; 95%CI 47.64 to 93.67). (**C**) The average number of female or male pups *per* year (from 2013 until July 2017) and by genotype. Statistics was performed by Kruskal-Wallis test, followed by Dunn’s multiple comparisons post-hoc test. Data for panels (**A**,**C**) were visualized utilizing box-and-whiskers plots in which the top and bottom lines represent the highest and lowest values whereas the height of the box, represents the interquartile range. The line in the middle of the box represents the median.

*Wdfy3*^+/*lacZ*^ mice, just as other transgenic models^18^, were born at Mendelian ratios (*Wdfy3*^+/*lacZ*^/WT ratio = 0.7 ± 0.2, mean ± SD; *n* = 518 mice born). *Wdfy3*^+/*lacZ*^ dams compared to WT ones had smaller litter sizes at both birth and weaning (decreased by about 20–25%; Fig. 3A). Several haploinsufficient pups died within the first postnatal weeks (Fig. 3A,B), resulting in lower-than-Mendelian ratios of survivors at weaning (Fig. 3C). Changing the mating scheme from haploinsufficient female × WT male to haploinsufficient male × WT female improved the litter size as well as their survival by >20% (Fig. 3A,B). Deficits in rearing success by *Wdfy3*^+/*lacZ*^ dams, compared to WT, were inferred from the wean-to-born ratio, which when calculated by *Wdfy3*^+/*lacZ*^ dams was significantly decreased (by 24%, *p* = 0.023; Fig. 3B) compared to either WT dams or published ratios for C57BL/6J^25^. While a significant loss of *Wdfy3*^+/*lacZ*^ pups was observed (Fig. 3C), *Wdfy3*^+/*lacZ*^ females appeared to survive at a greater extent than males at weaning (by 7–10 mice; *p* = 0.092; Fig. 3C). As a result, the proportion of females was higher in the post-weaned *Wdfy3*^+/*lacZ*^ group than in WT (62.7% vs. 54.3%, respectively; *p* = 0.018; Z-statistics 2.361; 95%CI = 55.52 to 69.49).

**Figure 4:**
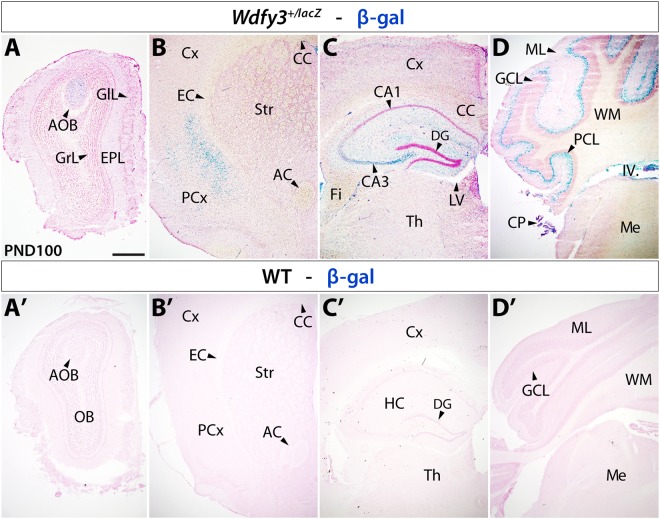
Wdfy3 β-gal reporter expression in *Wdfy3*^+/*lacZ*^ mice Top panel: Progressively more posteriorly located coronal brain sections of 3-m old (PND100) *Wdfy3*^+/*lacZ*^ mice reveal β-gal expression under *Wdfy3* control in brain areas specifically examined for mitochondrial dysfunction. Anteriorly (**A**), strongest expression can be seen in accessory olfactory bulb (AOB) and dispersed cells within external plexiform (EPL) and glomerular layers (GlL) of olfactory bulb (OB). In cortex (**B**,**C**) dispersed β-gal^+^ cells can be seen throughout, but condensed in the piriform cortex (PCx). Pronounced β-gal expression can also be detected in the hippocampal (HC) CA3 region (**C**), choroid plexus (CP), and cerebellar Purkinje cell layer (PCL, **D**). Bottom panel: WT brain processed for X-gal staining reveal absence of β-gal expression. Antero-posterior locations line up in (**A’**) with olfactory bulb (OB), (**B’**) striatum (Str), (**C’**) anterior hippocampus (HC), and (**D’**) cerebellum. Abbreviations: IV. fourth ventricle; AC, anterior commissure; AOB, accessory olfactory bulb; CC, corpus callosum; DG, dentate gyrus; EC, external capsule; Fi, fimbria; GCL, granular cell layer; GIL, glomerular layer; LV, lateral ventricle; MB, midbrain; Me, medulla; ML, molecular layer; Str, striatum; Th, thalamus; WM, white matter. Scale bar is 500 µm.

In previously reported findings^16^, we noticed cerebral lengthening and mild cortical abnormalities in *Wdfy3*-haploinsufficient mice at birth. To explore whether overt neuropathological abnormalities were observed in adult *Wdfy3*^+/*lacZ*^ mice, we examined brains by whole-mount imaging and in sections (Supplementary Fig. S1). While we noted a trend towards increased brain size in adult *Wdfy3*^+/*lacZ*^ mice, analysis of whole-mount brains by wet weight did not reveal significant differences between genotypes (Student’s *t*-test, *p* = 0.301). Further histological examination of hematoxylin-eosin-stained sections also did not reveal any differences in brain morphology between genotypes confirming that any perinatal structural anomalies are transient in nature and absent in adult life (Supplementary Fig. S1). To gain a better understanding of Wdfy3’s role in adult brain, we evaluated Wdfy3 expression by following the endogenous expression of the *lacZ*-reporter gene inserted into the *Wdfy3* locus through X-gal staining (Fig. 4A–D, top panels). X-gal staining confirmed that Wdfy3 was widely expressed in adult cortex and cerebellum as well as in other brain structures, with highest levels seen in accessory olfactory bulb, hippocampal CA3 region, lateral cortical aspects, and cerebellar Purkinje cell layer. Expectedly, X-gal staining on WT brain sections did not reveal any signal confirming that X-gal labeling was exclusively due to endogenous *lacZ* expression (Fig. 4A’–D’, bottom panels). Overall, a greater proportion of cells expressing Wdfy3 was visualized in cerebellum than cortex (Fig. 4B–D), consistent with the reported proteomic profile of Wdfy3 expression in WT mice (Wdfy3 expression cerebellum-to-cortex = 70-to-1^26^). These results are also consistent with *Wdfy3* expression data obtained in embryonic, perinatal and adult brains^14,18^ as well as in both neurons and glia^18^.

### Pathway analysis revealed that Wdfy3 is required for axon guidance, bioenergetics, mitophagy, translation, transport, nonsense-mediated decay, cytoskeleton remodeling and neurodevelopmental processes

**Figure 5:**
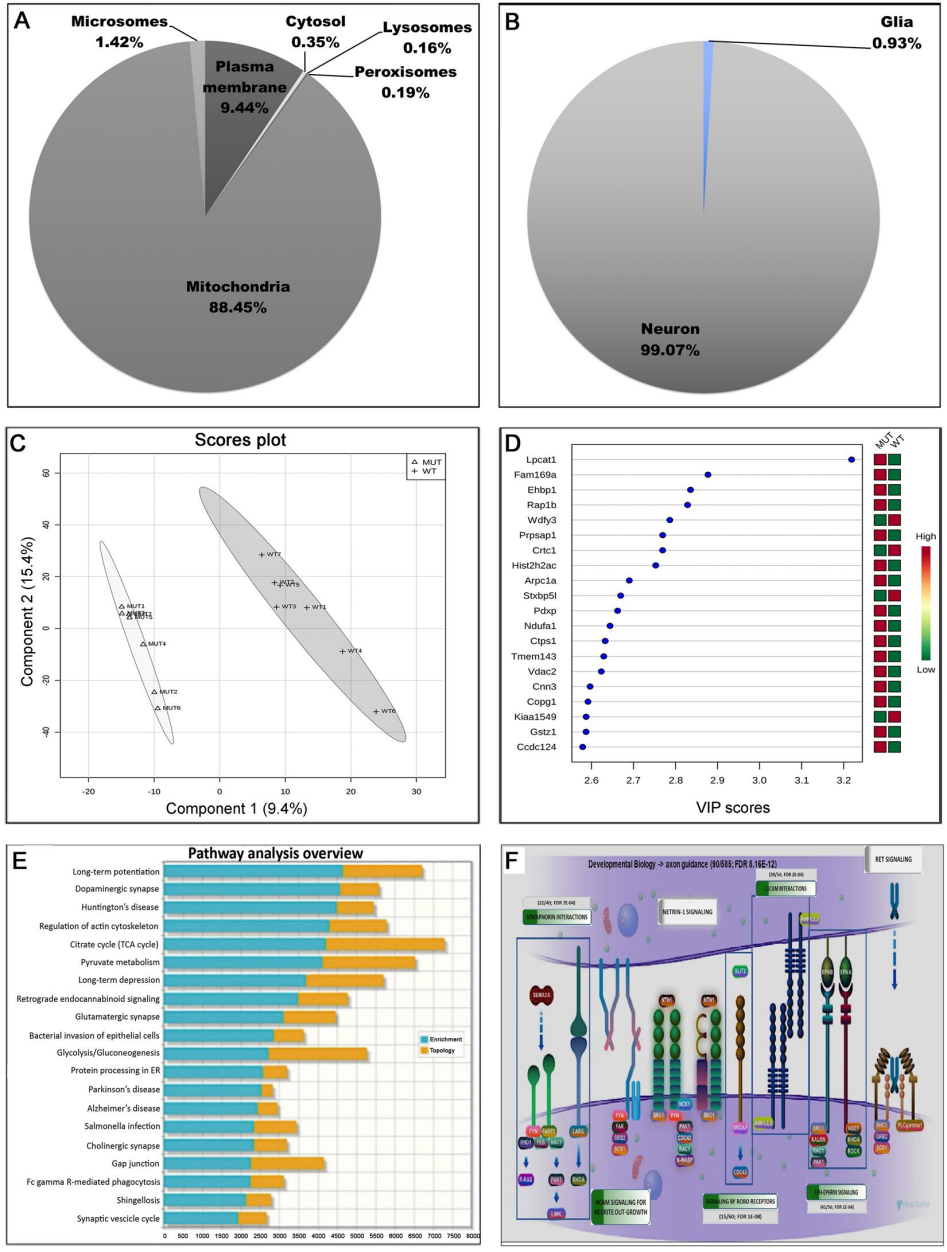
Pathway analysis based on cortical proteomic data from *Wdfy3-*haploinsufficient mice. (**A,B**) Purity of brain cortical mitochondria fractions is shown as percentage of mitochondrial-to-cytosolic proteins identified in each of these fractions by proteomics analysis. Proteomics was performed on mitochondrial fractions from cortex of seven 3-m old mice. The resulting proteins were filtered by using the gene ontology “cellular compartment” feature. The percentage of proteins from each indicated subcellular compartment (**A**) or cell type (**B**) was calculated. (**C**) Cortical mitochondrial proteins from both genotypes were identified by mass spectrometry and quantified by spectral counting. PLS-DA analysis of the two groups provided a visual interpretation of the complex proteomic datasets through scores plot that illustrated a suitable separation between the two genotypes. From this analysis, those proteins with VIP >0.8 were selected (only top 20 are shown in panel (**D**); the rest were included under Supplementary Dataset, “**PLS-DA”** tab). (**E**) Pathway over-representation analysis was performed by using those proteins with VIP score >0.8 and utilizing the KEGG database. (**F**) Image for “Axon guidance” (R-HSA-422475)^78^ obtained from pathway analysis of proteomics data performed with REACTOME (https://reactome.org). Only those with numerical values were significant; the enrichment (as a ratio of input proteins over total proteins in pathway) and the false discovery rate (FDR) are shown.

To understand the molecular consequences of Wdfy3 haploinsufficiency on Wdfy3 bound to membranes and scaffolding structures as well as to organelles, such as mitochondria, we performed untargeted proteomics on cortical mitochondria-enriched fractions from WT and *Wdfy3*^+/*lacZ*^ mice (*n* = 7 mice per genotype). The purity of the mitochondrial fraction was (on average) 88.45%, estimated by calculating the percentage of mitochondrial over cytosolic proteins identified in each of these fractions by proteomics (Fig. 5A). The contamination of the mitochondria-enriched fraction with other subcellular compartments was negligible (with the lowest at 0.16% for the lysosomes to the highest with 9.4% for the plasma membrane; Fig. 5A). Similarly, we found the mitochondrial fractions used in our analysis to be predominantly derived from neurons with minimal glial contribution (106-to-1 ratio; Fig. 5B).

Proteins (identified by mass spectrometry and quantified by spectral counting) in these mitochondrial fractions were used as input for partial-least-squares discriminant analysis (PLS-DA; Fig. 5C). This chemometric technique provides a visual interpretation of the complex proteomic datasets through an easily interpretable scores plot that in our study confirmed a suitable separation between the two genotypes. This technique also provided variable importance on projection (VIP) scores, from which only those with VIP >0.8 were used for further analysis (Fig. 5D; Supplementary Dataset). By utilizing the subset of proteins with VIP >0.8, pathway over-representation analysis was performed by using the REACTOME (Supplementary Dataset) or the KEGG (Fig. 5E; Supplementary Dataset) databases. This pathway analysis indicated that axon guidance, bioenergetics, mitophagy, translation, transport, nonsense-mediated decay, and cytoskeleton remodeling were the top dysregulated pathways along with those involved in neurodevelopment (Robo, ephrin, L1cam and semaphorin; Fig. 5F; Supplementary Dataset).

**Figure 6:**
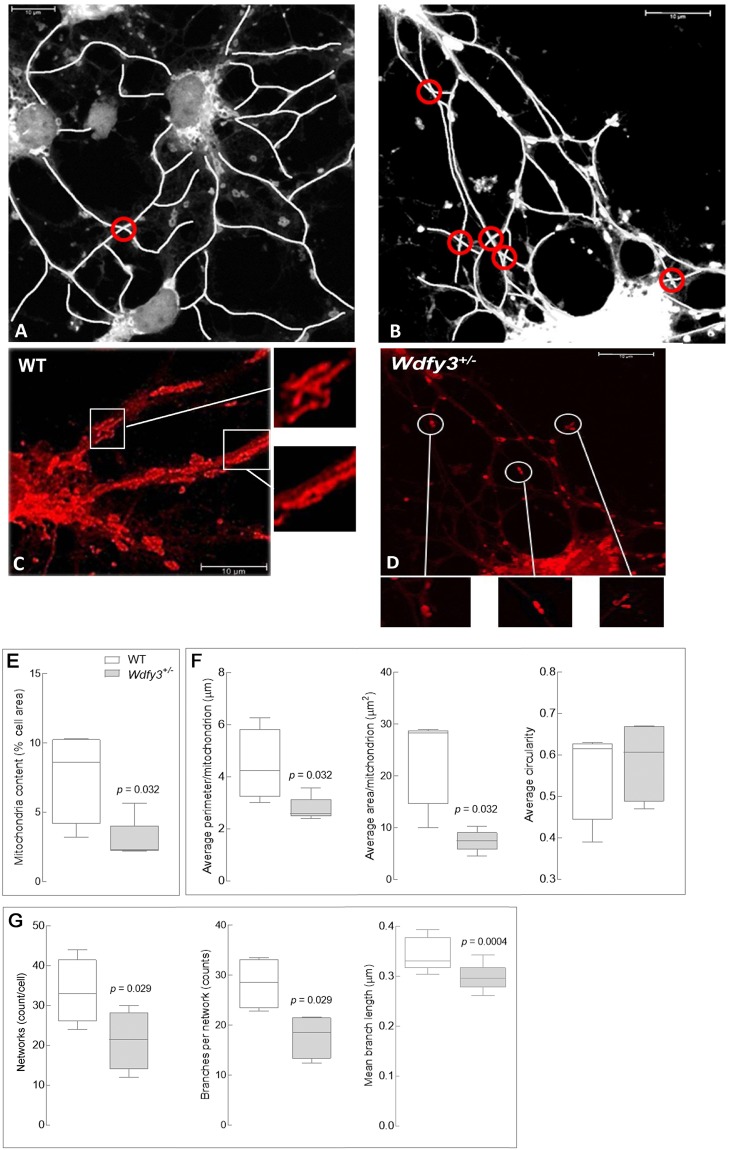
Mitochondrial morphology, distribution and projection pattern of cortical primary neurons in *Wdfy3*-haploinsufficient mice. Mitochondrial staining of primary cortical neurons from WT (**A**,**C**) or *Wdfy3*-haploinsufficient mice (**B**,**D**) was performed with MitoTracker and images recorded with an Olympus FV1000 confocal microscope at 60X magnification, as described in the Methods Section. A tubular, elongated pattern is evident in mitochondria from WT (**C**, insets) whereas *Wdfy3*^+/*lacZ*^ neurons show fewer, smaller mitochondria and budding protrusions (**D**, insets). Overlapping projections (panels A,B, circled in red) were visualized with ImageJ and their frequencies counted manually. (**E**,**F**) Quantification of the mitochondria content [percentage of cellular area covered by mitochondria, (**E**)] and mitochondrial morphology (**F**) were performed used a macro for ImageJ (see Methods). (**G**) Number of networks per cell and number of branches *per* network were evaluated with the MiNA macro (see Methods). Statistical analysis was performed with a 2-tailed Mann-Whitney test. Significance was considered with *p* values < 0.05.

An important neurodevelopmental phenomenon apparently connected to Wdfy3 involves mechanisms that help dendrites originating from a single neuron practice self-avoidance in order to be arranged in spatially non-overlapping patterns, a process requiring contact-dependent recognition and repulsion. Slit2 and its receptor Robo2 have recently been implicated in self-avoidance of cerebellar Purkinje cell dendrites, in which their deletion leads to excessive dendrite self-crossing without affecting arbor size and shape^27^. Consistent with the observed down-regulation of the Robo/Slit pathway, the frequency of neurite self-crossing in cortical neurons cultured from *Wdfy3*^+/*lacZ*^ mice was significantly higher (>2–3/cell) than WT (<1/cell; Fig. 6A,B; *p* = 0.038). Notably, this pattern resembles the autophagic deficits underlying spine pruning defects observed in both brains from ASD-affected human subjects as well as *Tsc2*^+/−^ mice^28^.

### Wdfy3 is critical for mitophagy, mitochondrial function and distribution in neuronal projections

Synaptic function and plasticity rely on mitochondrial function and efficient trafficking of mitochondria to neuronal projections^29^. The attachment of mitochondrial organelles to microtubule-based motor proteins contributes to anterograde trafficking to provide energy and metabolic turnover needed to sustain neurotransmission and calcium regulation^22^. The opposite mechanism, retrograde transport of compromised mitochondria, is essential for their clearance *via* mitophagy^21^. Considering the role of Wdfy3 in autophagy and the critical role of mitochondrial fatty acid β-oxidation in sustaining neural stem cell self-renewal^20^ we sought to assess mitochondria dynamics (i.e., distribution and morphology) and bioenergetics (i.e., ATP generation by mitochondria) in *Wdfy3*^+/*lacZ*^ mice.

To visualize mitochondrial dynamics, primary cortical neurons from cortex of sex- and age-matched WT and *Wdfy3*^+/*lacZ*^ mice were stained with MitoTracker, a dye that accumulates and is retained by polarized mitochondria^30^. Mitochondria from WT cortical neurons were elongated and tubular, extending from soma to projections (Fig. 6C). In contrast, cortical neurons from haploinsufficient mice showed fewer polarized mitochondria (Fig. 6D). This observation is consistent with other microscopy-based assessments, notably the smaller total mitochondrial area *per* cell (Fig. 6E), as well as the average perimeter and area *per* mitochondrion; Fig. 6F). Further, in *Wdfy3*^+/*lacZ*^ neurons, mitochondria were organized within more sparse networks with fewer, shorter branches (Fig. 6G**)**, indicating issues with mitochondrial transport along projections. Independent evaluation of mitochondrial mass (not related to polarization) was performed by assessing citrate synthase activity in cortex from WT and haploinsufficient mice (Supplementary Table S1). This activity was significantly higher in *Wdfy3*^+/*lacZ*^ cortex vs. controls; however, the difference was only by ~9%, thus supporting the idea that the decreased polarized, functional mitochondria was not the result of lower content of mitochondria.

**Figure 7:**
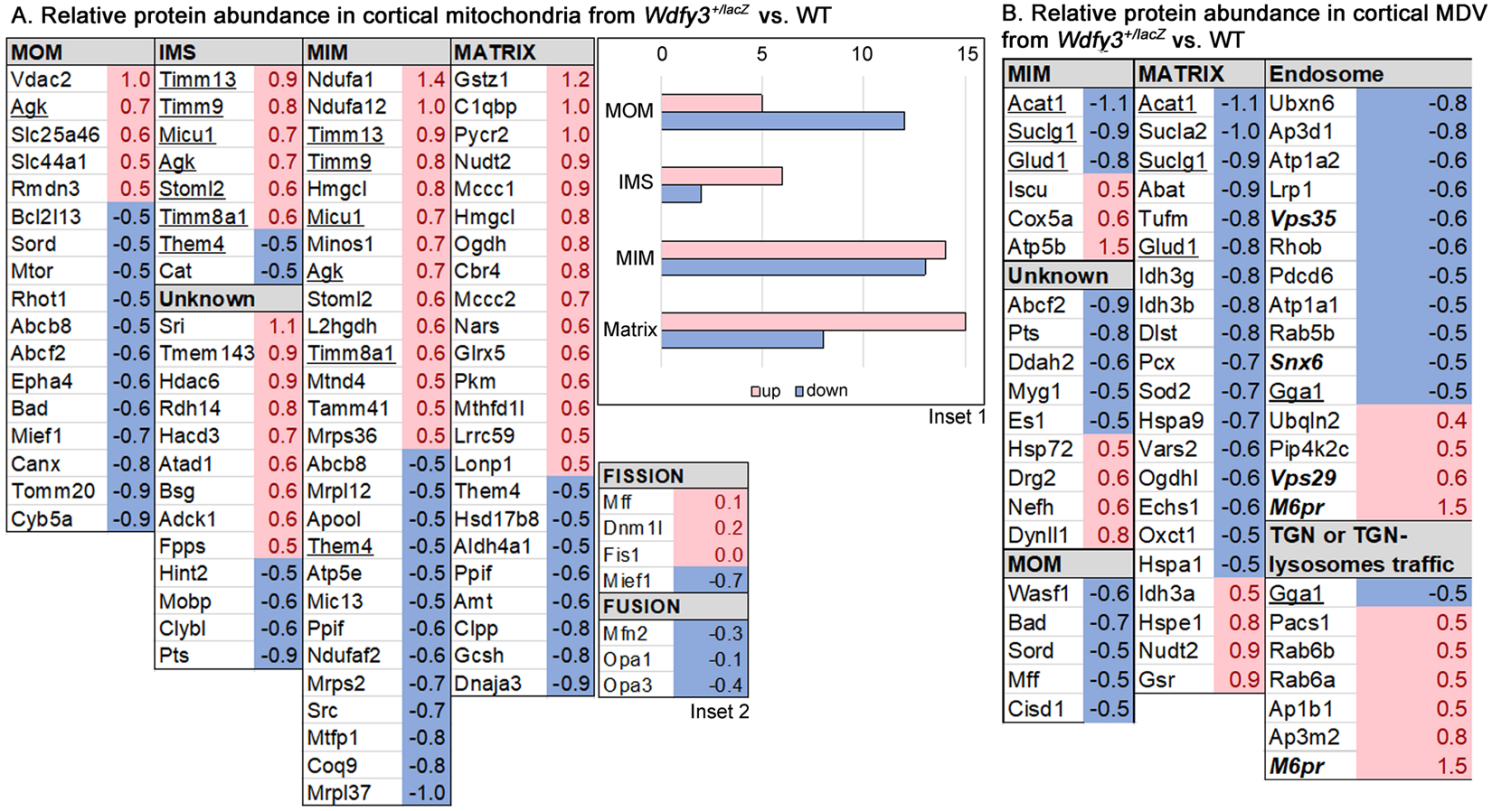
Characteristics of cortical mitochondria and MDVs from W*dfy3*-haploinsufficient mice vs. WT mice. (**A**) Relative abundance of mitochondrial proteins in cortical mitochondrial subcompartments from Wfdy3^+/*lacZ*^ vs. WT. Proteomics data obtained from cortical mitochondria-enriched fractions from WT and *Wdfy3*^+/*lacZ*^ mice (3-m old; *n* = 7/genotype) were filtered as described in the text and Methods. The fold changes for these proteins were calculated by using the log2 of the value for haploinsufficient over that of the WT for each protein. Only those with at least changes ± 0.5 fold are shown. Proteins with higher or lower abundance in *Wdfy3*^+/*lacZ*^ vs. WT are shown in red or blue, respectively. The subcellular allocation was performed by following the gene ontology “cellular compartment”. Inset 1: Number of mitochondrial proteins with higher or lower abundance in *Wdfy3*^+/*lacZ*^ vs. WT *per* submitochondrial compartment. Inset 2: Proteins detected in cortical mitochondria associated with the terms fusion or fission. (**B**) Relative abundance of proteins in cortical MDV from *Wfdy3*^+/*lacZ*^ vs. WT. Proteomics data obtained from MDV-enriched fractions from WT and *Wdfy3*^+/*lacZ*^ mice (3-m old; *n* = 7/genotype) were filtered as indicated under panel A. The data obtained under panel B was filtered by cellular compartments. Only those proteins showing changes ± 0.5 are shown. Abbreviations: MOM, mitochondrial outer membrane; IMS, intermembrane space; MIM, mitochondrial inner membrane. Unknown stands for mitochondrial proteins with no determined or specific submitochondrial compartment.

While mitochondrial depolarization is a key event for triggering Pink1- and Parkin-dependent mitophagy^31^, the accumulation of depolarized mitochondria in *Wdfy3*^+/*lacZ*^ was suggestive of halted or defective mitophagy. Moreover, some mitochondria in primary cortical neurons from haploinsufficient mice exhibited budding protrusions (Fig. 6C,D**)**, sometimes surrounded by smaller mitochondrial particles. These structures were reminiscent of mitochondria-derived vesicles (MDV) detected in an autophagic process termed micromitophagy that occurs independently of Pink1- and Parkin-dependent mitophagy^31^. Micromitophagy (also known as Type 3 mitophagy) includes the release of cargo-selective vesicles that bud off mitochondria independently of the mitochondrial fission machinery. Based on the most recent reports on MDV, these vesicles differ from mitochondrial fragments by at least three distinct criteria: (i) the nature of the cargo incorporated within vesicles; (ii) formation through a Drp1-independent manner; and (iii) morphology, in that they have a limited diameter of <200 nm containing either a single outer membrane or a double membrane-bound structure lacking cristae^32,31^. Proteomics analyses performed on both mitochondria-enriched fractions and MDV-enriched fractions revealed that cortical mitochondria from *Wdfy3*^+/*lacZ*^ were enriched with mitochondrial proteins located at the intermembrane space, inner membrane and matrix and mostly depleted of proteins at the outer membrane (Fig. 7A, inset 1). The ratio of the sum of all mitochondrial proteins identified in the MDV fraction in haploinsufficient mice relative to WT was 2.1 ± 0.2 (mean ± SE; *n* = 109 proteins) suggesting that cortical neurons had 2-fold enrichment of MDVs compared to WT. Furthermore, the MDV fraction was enriched in specific mitochondrial proteins, as well as a few proteins from the retromer complex and from endosomes as observed by others^33,34^ (Fig. 7B), and also enriched in some, but not all, subunits of Complex IV, Complex V and isocitrate dehydrogenase, as previously reported^35,36^ (Fig. 7B). Taken together, the presence of smaller and budding mitochondria (Fig. 6), decreased mitochondrial envelope cargo in the mitochondrial fraction (Fig. 7), an estimated size of MDV of <200 nm (Fig. 6), and cargo composition of the MDV fraction (Fig. 7) are highly indicative of the activation of micromitophagy. Furthermore, the lack of differences in the abundance of fission proteins between the two genotypes in cortical mitochondria (Fig. 7A, inset 2), except Mief1, as well as in the MDV-enriched fraction (Fig. 7B) are indicative of a process that took place without significant input from the fusion-fission machinery. The lower abundance of the mitochondrial dynamics protein of 51 kD (Mief1) in the cortical mitochondrial fraction (Fig. 7A, inset 2) suggests that there might be down-regulation of the recruitment and association of Drp1 to the mitochondrial envelope as this protein is a key factor at regulating Drp1 GTPase activity as well as Drp1 oligomerization^37^. An overall assessment of these results is consistent with defective mitophagy being partly abrogated by enhanced micromitophagy.

**Figure 8:**
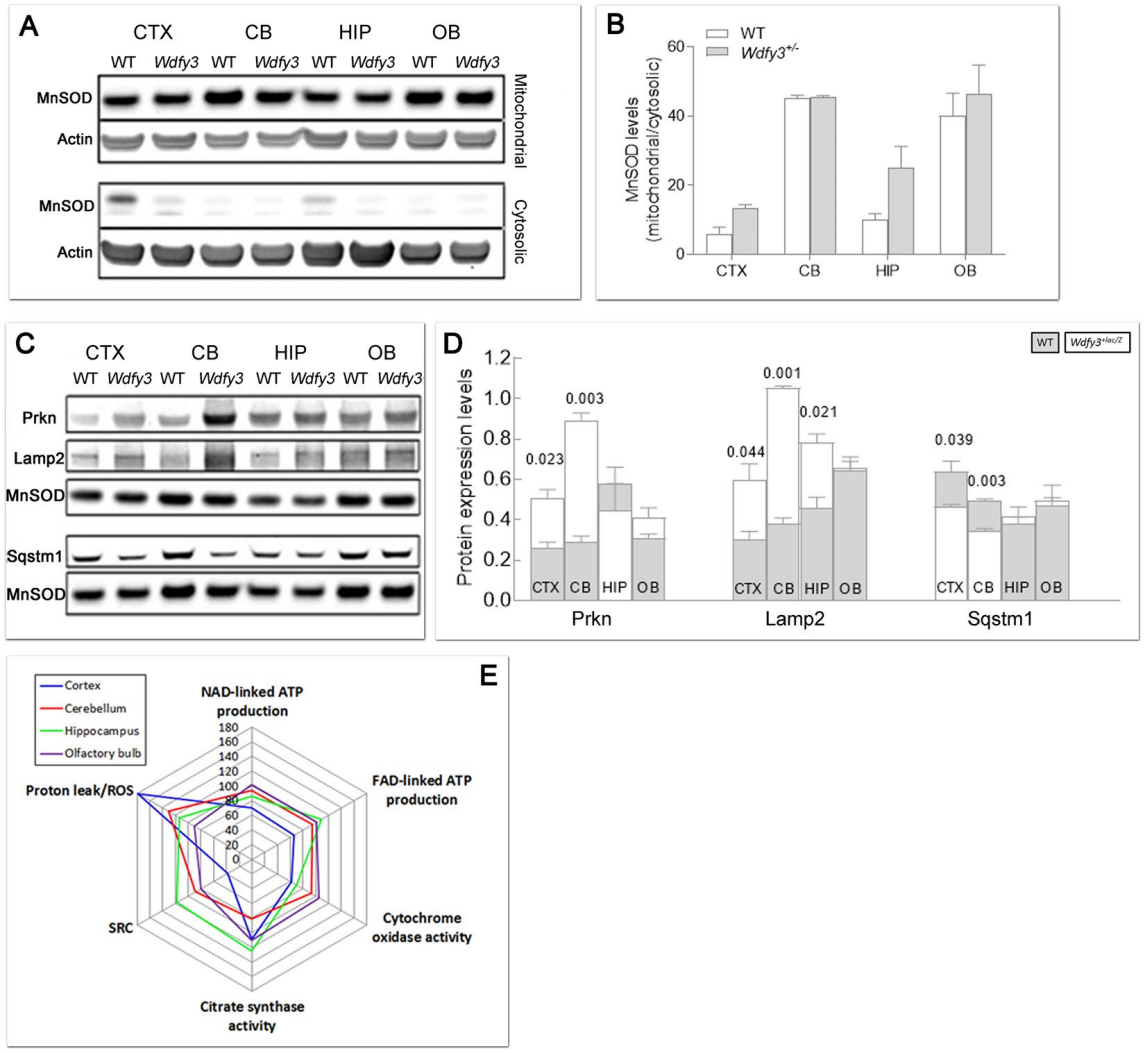
Expression of autophagy and mitophagy-related proteins and bioenergetics outcomes in brain regions from W*dfy3*-haploinsufficient mice. (**A,B**) Degree of mitochondrial enrichment *per* each brain area. Shown are representative Western blots (**A**) and densitometry (**B**) of MnSOD and actin as markers of, respectively, mitochondrial and cytosolic compartments, for evaluation of mitochondria purity in each brain region. Statistical analysis was performed with the Mann-Whitney test between WT and W*dfy3* mice for each brain area. (**C**,**D**) Representative Western blots (**C**) and densitometry (**D**) of mitophagy-related proteins (Parkin, Lamp2, Sqstm1) in mitochondrial-enriched fractions obtained from cortex (CTX), cerebellum (CB), hippocampus (HIP), and olfactory bulb (OB) of WT (gray bars) and W*dfy3*-haploinsufficient mice (white bars). Full-length Western blot images are shown in Supplementary Fig. S4. Data are shown as mean ± SEM of samples ran in duplicates (*n* = 3 WT and 5 *Wdfy3*^+/*lacZ*^). Statistical analysis was performed with the Mann-Whitney test between WT and W*dfy3* mice for each brain area. MnSOD was used as mitochondrial loading control. The protein expression of other mitophagic proteins (Pink1, Mfn2, Vdac and LC3I and LC3II) is shown in Supplementary Fig. S2. (**E**) ATP-driven oxygen uptake rates were evaluated in mitochondria-enriched fractions from the indicated brain regions from mice of each genotype. Substrates utilized for driving the oxygen uptake in phosphorylating mitochondria in the presence of ADP were malate-glutamate (NAD-linked ATP production) and succinate (FAD-linked ATP production). Complex IV or cytochrome *c* oxidase activity (CCO) is reported as a marker of mitochondrial inner membrane whereas citrate synthase activity (CS) as a marker of mitochondrial matrix. All values were expressed as percentages of control. All *p*-values calculated using the 1-tailed *t*-test. Values were taken from Supplementary Tables S1–S4. The number of mice utilized were *n* = 11 and 16 respectively for WT and *Wdfy3*^+/*lacZ*^ cortex and cerebellum, *n* = 3 and 5 respectively for WT and *Wdfy3*^+/*lacZ*^ hippocampus and olfactory bulb (Supplementary Tables S1–S4). A spider chart was used to plot the various mitochondrial outcomes to facilitate their visualization and comparison across brain regions. The spider plot consisted of a sequence of 5 equi-angular spokes or radii representing each of the mitochondrial outcomes evaluated. The data length of a spoke is proportional to the magnitude of the outcome relative to the maximum magnitude of all variables across all data points (i.e., 180). For each brain region, a line was drawn connecting the data values for each spoke, resulting in a star-like appearance.

To provide further and independent experimental support for these conclusions, the expression of key markers of mitophagy and autophagy was assessed by Western blotting in mitochondria-enriched fractions from cortex, cerebellum, hippocampus and olfactory bulb from WT and *Wdfy3*^+/*lacZ*^ mice. Mitochondrial enrichment of each tested brain region was also assessed by Western blot analysis (Fig. 8A,B). As judged by the mitochondrial-to-cytosolic MnSOD ratio, mitochondrial enrichment was 30 ± 6-fold (mean ± SEM considering all brain areas and genotypes), regardless of the genotype, with an optimal recovery based on the minimal fraction of MnSOD that was detected in the cytosolic fraction (MnSOD levels normalized to actin = 0.04 ± 0.02-fold). These ratios were 12-fold higher and 25-fold lower, respectively, than accepted values in the literature for the enrichment of mitochondrial marker enzymes in the mitochondrial fraction (at least 2.5) and depletion of other fractions (<1^38^).

**Figure 9:**
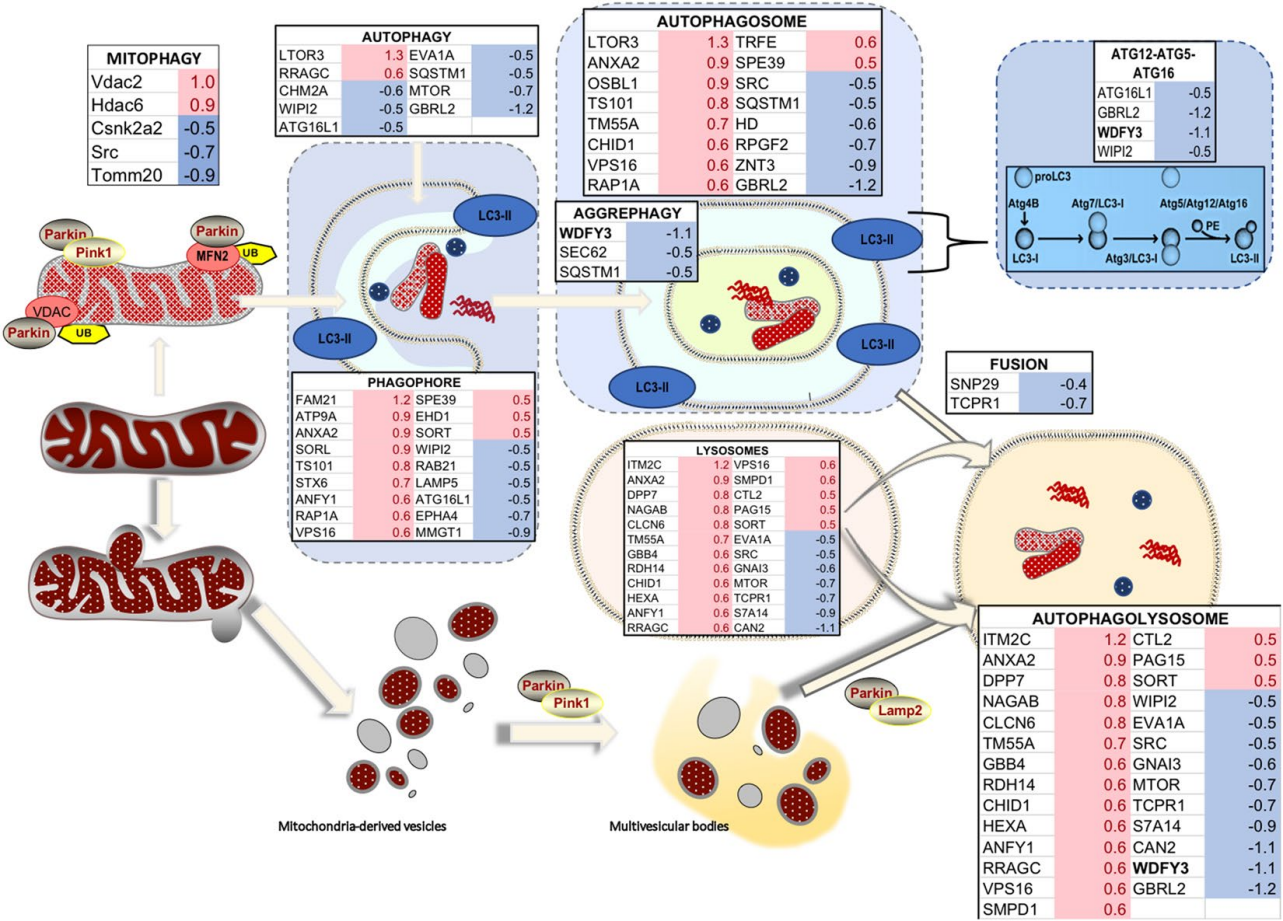
Differential expression of proteins involved in autophagy and mitophagy in brain of WT and *Wdfy3* mice. Proteomics was performed on cortical mitochondria-enriched fractions from WT and *Wdfy3*^+/*lacZ*^ mice (*n* = 7/genotype). Proteomics data were filtered as described in the Methods and main manuscript sections. The fold changes for these proteins were calculated by using the log 2 of the value for haploinsufficient over WT for each feature. Only those with 0.5 ≤ log2 FC ≤ −0.5 are shown. Upon Pink1-dependent recruitment of Parkin to depolarized mitochondria (mitophagy), the defective organelles are internalized within the autophagosome and degraded upon fusion of the autophagosome with lysosomes. Proteins involved in aggrephagy (to clear damaged, unfolded proteins shown as red, wavy structures), as well as ATG12-ATG5-ATG16l complex, were present at lower levels in mitochondria-enriched fractions from cortex of *Wdfy3*-haploinsufficient mice vs. WT. However, proteins involved in phagophore and autophagosome formation seemed enriched in these fractions (~2-fold), but accompanied by a decreased level of proteins mediating the fusion of autophagosomes with lysosomes. An autophagic independent degradation pathway exists, through the formation of mitochondria-derived vesicles and subsequent fusion with lysosomes. In red are shown upregulated proteins, in blue downregulated ones.

Consistent with the concept of halted mitophagy and enhanced micromitophagy, Pink1 (required for mitophagy activation *via* Parkin-phosphorylation), Mfn2 and Vdac (substrates of Parkin-mediated ubiquitination), as well as LC3I and LC3II levels (in which an increased LC3II-to-LC3I ratio is considered a marker of autophagy activation) were not significantly different between genotypes (Supplementary Fig. S2). But the expression levels of Parkin (Fig. 8C,D) and Lamp2 (a lysosomal marker; Fig. 8C,D) were significantly increased in mitochondria-enriched fractions of haploinsufficient mice (Parkin by 2- and 3-fold in cortex and cerebellum, respectively; Lamp2 by 2-fold in cortex and hippocampus, and by 3-fold in cerebellum) with a lower recruitment of Sqstm1/p62 to mitochondria (by 27% and 30% of WT in cortex and cerebellum, respectively; Fig. 8C,D). These results were further confirmed by filtering the mitochondrial proteomics data by keywords related to mitophagy and autophagy (Fig. 9). Of note, the relatively higher levels of Parkin in cortex and cerebellum in haploinsufficient mice vs. WT (Fig. 8B,C) were consistent with the reported dependence of the formation of MDVs on Parkin levels in HeLa and U2OS cells^36^; this and our results are in contrast with the previously reported suppression of MDVs by high levels of Parkin^39^. This discrepancy is likely due to differences in cell type (neurons vs. immune cells), effectors (Wdfy3 haploinsufficiency vs. immune triggers) and level of Parkin (about 2-fold, this study, vs. GST-tagged Parkin overexpression).

To evaluate whether Wdfy3-mediated effects on mitophagy and the ensuing activation of micromitophagy had any impact on bioenergetics, the capacity to generate ATP by mitochondria from WT and haploinsufficient mice was evaluated by using NAD^+^- and FAD^+^-linked substrates (namely malate-glutamate and succinate, respectively) in mitochondria-enriched fractions from cerebral cortex, cerebellum, hippocampus and olfactory bulb from adult *Wdfy3*^+/*lacZ*^ mice and sex- and age-matched controls (Fig. 8E and Supplementary Tables S1–S4). In addition, the spare respiratory capacity^29^, i.e. the extra ATP produced through OXPHOS in case of a sudden increase in energy demand, and oligomycin-resistant oxygen uptake (indicative of reactive oxygen species production and/or mitochondrial membrane proton leakiness) were also assessed. The activities of Complex IV (also known as cytochrome *c* oxidase or CCO) and citrate synthase (CS)—markers of mitochondrial inner membrane and matrix, respectively—were used to estimate the cristae-to-matrix ratio (CCO/CS; adapted from other reports^40^).

In *Wdfy3*-haploinsufficient mice, mitochondria from cortex and cerebellum presented the most significant deficits in all outcomes tested, followed by hippocampus (only one outcome affected; Fig. 8E and Supplementary Tables S1–S3), whereas olfactory bulb did not show any deficiencies (Fig. 8E and Supplementary Table S4). In cortex and cerebellum, these deficits entailed lower ATP production with both NAD^+^- and FAD^+^-linked substrates (by 23% with malate-glutamate in both brain areas, and by 27% and 23% with succinate in cortex and cerebellum, respectively; Fig. 8E and Supplementary Tables S1 and S2). We also noted lower CCO activity, as well as a decreased spared respiratory capacity (SRC) and increased reactive oxygen species (ROS)/proton leak. Overall, the most consistent deficit across brain regions (excluding olfactory bulb) was a decrease in cristae density (decreases by 35%, 21% and 33% of the CCO/CS ratio for cortex, cerebellum and hippocampus, respectively). The magnitude of this deficit was comparable to the overall decreased OXPHOS capacity in cortex and cerebellum. The decreases in ATP production (sustained by NAD^+^- or FAD^+^-linked substrates), CCO activity and spared respiratory capacity observed in cortex of haploinsufficient mice did not seem to be associated with a consistent decreased levels of mitochondrial respiratory chain protein complexes, as some of them (Ndufa1, Ndufa12, ND4) showed higher and others (Atp5e, Ndufaf2, Coq9) lower levels than WT (Fig. 7A). As indicated before, although a statistically significant difference in the activity of the matrix marker citrate synthase was noted in cortex and cerebellum of *Wdfy3*^+/*lacZ*^ mice relative to WT, the extent of this difference argues against its biological relevance *in vivo* (9% for both brain areas). Thus, this suggests that variations in mitochondrial biogenesis are not relevant to the phenotypes we have observed, and instead emphasizes the accumulation of damaged proteins within mitochondria as a primary mechanism preventing their normal assembly into super-complexes and ATP production.

**Figure 10:**
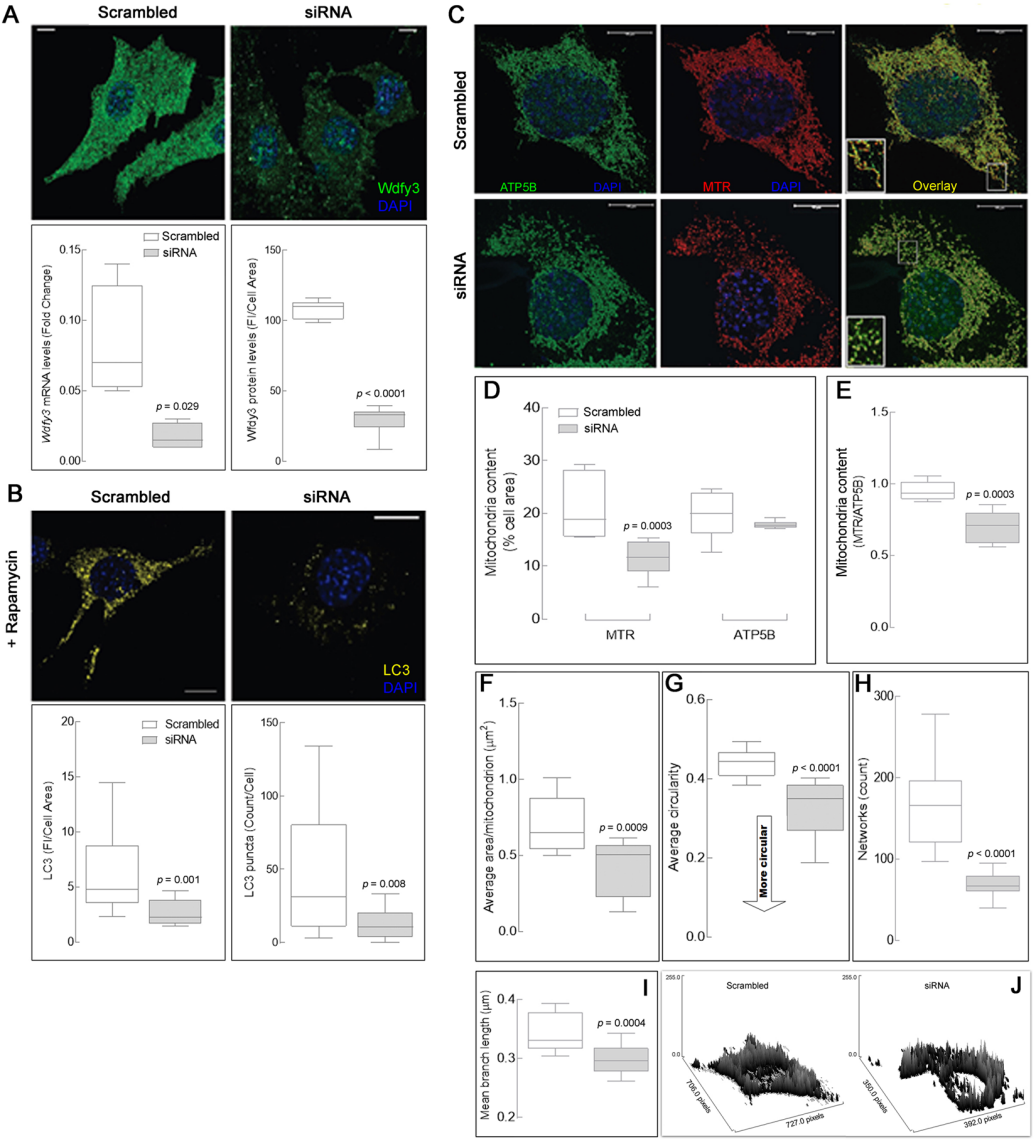
Dampening of autophagy activation in *Wdfy3*-deficient neuronal progenitor cells is accompanied by decreased mitophagy. (**A**) Wdfy3 immunofluorescent labeling in striatal neuronal progenitor cells (NPCs) shows ubiquitous expression (scrambled). A significant down regulation of Wdfy3 was observed in siRNA-transfected cells. Quantification of Wdfy3 protein levels was based on fluorescent signal intensity normalized by cell area. Levels of *Wdfy3* mRNA expression were assessed by qPCR normalized to *Gapdh* mRNA. (**B**) The effect of rapamycin (20 nM) addition was evaluated by quantifying LC3 immunostaining normalized by cell area, as well as the number of LC3 puncta per cell. Statistical analyses were performed by two-tailed Mann-Whitney test. (**C)** Total mitochondrial mass was visualized in scrambled and siRNA striatal cells by staining with antibodies to ATP5B (green) whereas polarized mitochondria were stained with MitoTracker (red). Co-localization of functional and total mitochondria is shown in yellow (overlay). Insets show details of the mitochondrial morphology and co-localization of ATP5B and MitoTracker in the two genotypes. Red channel intensity has been increased for siRNA-transfected cells, due to the low MitoTracker fluorescence signal emitted by non-polarized mitochondria. Scale bars = 10 µm. **Panels (D–H**). Mitochondrial content (**D**) was quantified as the cell area occupied by mitochondria (in %). Mitochondrial footprint (**E**) was expressed as functional mitochondria (area) normalized by total mitochondria area. (**F**) Average area per mitochondrion in scrambled- and si-RNA striatal cells. Mitochondrial morphology (**G**) was reported as circularity index, with mitochondria having a more tubular shape being closer to a value of 1. All measurements shown in panels D,G were performed using a macro for ImageJ (see Methods). (**H**,**I**) Assessment of the mitochondrial network integrity in scrambled- and siRNA striatal neurons was performed with the MiNA macro for ImageJ and reported as number of networks per cell (**H**) and mean branch length (**I**). Statistical analysis for panels D–I was performed with a 2-tailed Mann-Whitney test. (**J**) Surface plots representing levels and mitochondrial distribution in scrambled (left) and siRNA (right) striatal cells were obtained with the ImageJ feature “surface plot”. All the box plots shown in panels F–I were obtained analyzing confocal images of mitochondria stained with MitoTracker.

The above results obtained *in vivo* were supported by *in vitro* findings utilizing a different, yet relevant, biological model. This model consisted of siRNA-mediated *Wdfy3* knockdown in murine neuronal (striatal) progenitor cells (NPCs), which resulted in *Wdfy3* levels reduced by >70%. Concomitant gene expression levels, evaluated by qPCR were reduced to 22% of control levels and protein expression by immunocytochemistry to 28% of levels seen in scrambled-RNA-treated NPCs (Fig. 10A). Treatment with the mTOR inhibitor and autophagy activator rapamycin increased LC3 staining (a marker of autophagy) and number of LC3^+^ puncta in control neurons, a response that was significantly blunted in Wdfy3-deficient neurons (by 70%; Fig. 10B). Thus, as seen *in vivo*, in this cellular model the LC3-dependent step, shared by both autophagy and mitophagy, was halted (Supplementary Fig. S2).

Because mitochondria of cultured *Wdfy3*^+/*lacZ*^ primary cortical neurons displayed defective morphology and distribution, we used confocal microscopy to evaluate total mitochondrial mass (assessed by the abundant β-subunit of the mitochondrial F_o_F_1_-ATPase or ATP5B) and visualize polarized mitochondria following MitoTracker staining in NPCs (Fig. 10C). Total mitochondrial mass in Wdfy3-deficient NPCs was comparable to controls (Fig. 10C), whereas the mass of polarized mitochondria was decreased by 30–40% in Wdfy3-deficient NPCs (Fig. 10C,D). Comparable to our observations in cortical primary neurons from haploinsufficient mice (Fig. 6D,F), but more pronounced due to the almost complete loss of Wdfy3 induced by siRNA knockdown, mitochondria from *Wdfy3*-silenced NPCs were smaller (Fig. 10E,F), less tubular in shape (Fig. 10G), and as part of a disrupted network (less networks with smaller branches; Fig. 10H,I) compared to the more tubular and evenly distributed mitochondria of scrambled RNA-treated control NPCs. Furthermore, most Wdfy3-deficient NPC mitochondria had a noticeably perinuclear distribution, with fewer organelles localized in the cellular periphery and projections (Fig. 10J). Taken together, these results were highly indicative of an imbalance between antero- and retrograde trafficking of mitochondria along neuronal projections as observed in some neurodegenerative diseases such as Parkinson’s disease^41^ and fragileX-associated tremor and ataxia syndrome^42^.

Overall, our results pointed to a critical role for Wdfy3 in maintaining mitochondrial health and bioenergetic properties. The effects are driven by a pathological accumulation of defective mitochondria with an overall diminished OXPHOS capacity (including lower fatty acid β-oxidation) in *Wdfy3* haploinsufficiency.

## Discussion

*Wdfy3* encodes a phosphatidylinositol 3-phosphate-binding protein that functions as a master conductor of autophagy and aggrephagy (i.e., clearance of protein aggregates)^13,18^. Through its role in autophagy or other yet undescribed properties, Wdfy3 exerts profound effects during embryonic development. These include the regulation of division and migration of neural progenitors, which depending on heterozygous or homozygous mutations, result in excess mitoses of radial glial cells and increased neurogenesis^16^. In regards to neurodevelopment, *Wdfy3* has emerged as a risk gene in ASD^10–12,43^, likely through its role in developmental neurogenesis and/or possibly through additional functions it may exert later in life. Incidentally, homozygous mutant mice die perinatally, but heterozygotes survive to adulthood. The link between Wdfy3 (as an extension for autophagy) and psychiatric disorders has received support by recent findings of brain-specific downregulation of beclin1 in schizophrenic individuals^44^, while indirect evidence was provided by studies showing that lithium, widely used of the treatment of bipolar disorder^45^, along with commonly used antidepressants, induce autophagy^46^. Parkin and Pink1, two proteins implicated in mitophagy, are associated with familial Parkinson’s disease, in which sustained dysregulation of neuronal mitochondrial quality control can lead to neurodegeneration^47^.

Mitochondrial homeostasis is the result of a fine balance between biogenesis and clearance by autophagy, raising the possibility of a close association between autophagy dysfunction and accumulation of defective mitochondria. At early stages of life, such dysfunction may affect neurodevelopment or over time may lead to neurodegeneration, ensuing in associated psychiatric disorders. Consequently, we evaluated whether Wdfy3 was implicated in brain mitochondrial quality control through mitophagy. In the process, we confirmed that deficits in Wdfy3 are associated with a decline in Atg12-Atg5-Atg16l complex (as previously shown^13^) suggesting that *Wdfy3* haploinsufficiency alters the progression of Atg3/LC3I to Atg5/Atg12/Atg16L1/Wdfy3, likely preventing LC3I lipidation. This is supported by the constant levels of LC3I expression present in all tissues of both genotypes (Supplementary Fig. S2), the lack of genotype differences in the expression of LC3II, Atg7, Atg3, or Atg4b, but the lower levels of Atg16l1, GBRL2, and WIPI2 (Fig. 9). Our findings are in line with an autophagic role reported for Wdfy3^18^ and another WDR-containing protein, WDR40-47^17^, but expanding the role of Wdfy3 to include the selective clearance of defective mitochondria (mitophagy) and mitochondria trafficking.

Decreased mitophagy and autophagy activity (Figs 8–10**)** along with the presence of MDV (Figs 6–7**)**, as well as higher levels of Parkin and Lamp2 (Fig. 8), accompanied by a relatively modest decrease in overall OXPHOS capacity (~70% of WT; Fig. 8E) are consistent with the activation of micromitophagy as an apparent compensatory cellular response to partially clear some of the damaged mitochondria. In the presence of Wdfy3 haploinsufficiency, the accumulation of defective mitochondria indicates that diminished mitochondrial quality control could not be fully compensated for by the activation of micromitophagy. This can be interpreted as Wdfy3 being also required for micromitophagy, or alternatively, that micromitophagy, even at its full capacity, cannot abrogate all mitochondrial clearance deficits. The results obtained with primary cortical neurons are consistent with the ones obtained with NPCs, as partial (by ~50%; Fig. 6) or nearly total (by ~70%; Fig. 10) loss of Wdfy3 results in accumulation of damaged (depolarized) mitochondria with aberrant morphology and distribution.

At the molecular level, deficits in bioenergetics entailed lower OXPHOS capacity and Complex IV (normalized by CS) at comparable levels in both cortex and cerebellum. Notably, Complex IV (CCO) is highly reliant on the mitochondrial inner membrane lipid composition as illustrated by biological models with defects in cardiolipin synthesis (ceramide synthase 2 KO mice^48^ and Barth syndrome^49^). As autophagy is triggered under starving conditions to favor the recycling of proteins as well as lipids, a block in mitophagy or autophagy may challenge this process, compromising the lipid composition of cell membranes, including the inner mitochondrial membrane, and the stabilization of the respiratory chain super-complexes. This may explain both the lower overall OXPHOS capacity and CCO/CS ratio in cortex and cerebellum from haploinsufficient mice. Consistent with this premise, *Drosophila* mutants for the *Wdfy3* homologous *blue cheese* gene (*bchs*) showed an imbalance in ceramide metabolism with altered autophagy^50^. The differences in severity of mitochondrial defects between cerebellum and cortex vs. hippocampus might be explained by the relatively shorter maturation period and slower neuronal migration and dispersion pattern reported for hippocampus^51^, in addition to the higher turnover of hippocampal cells due to adult neurogenesis. Whatever the causes for differential outcomes in mitochondrial bioenergetics may be, neuron-to-glia ratios appear not to be relevant in this context, as they differ widely between cortex (1:4 ratio) and cerebellum (4:1 ratio)^52^.

In parallel with mitophagy, deficits in Wdfy3-affected mitochondrial dynamics (distribution and morphology) in murine brains; similarly, *in vitro* analysis in *Wdfy3*-silenced NPCs showed disrupted mitochondrial dynamics, with an accumulation of damaged mitochondria due to impaired clearance by LC3-dependent autophagy. When the proteomic data were analyzed against the KEGG database, pathways affected in Wdfy3 haploinsufficiency were shared with those related to neurodegenerative diseases (ALS, Parkinson’s, Huntington’s, Prion diseases) as well as those involved in vesicular transport, and dopaminergic and GABAergic synapses (Fig. 5E,F), pointing not only to decreased mitophagy, but also to defective intracellular transport (likely including mitochondria) and neuron development and/or organization. In addition, pathways inherent to OXPHOS, including fatty acid β-oxidation, were noted as down-regulated (Supplementary Dataset under “pathways” and “biological processes” tabs). Consistent with the notion that fatty acid β-oxidation is key in controlling the NSC-to-IPC transition in the mammalian embryonic brain^20^, a lower abundance of proteins associated with neuron differentiation was observed in *Wdfy3*^+/*lacZ*^ mice (i.e., spectrins, voltage-dependent channels; Supplementary Dataset under “biological processes” tab). These findings are consistent with a recent report utilizing *Wdfy3* transgenic mice (gene trap and constitutive knockout) indicating a central role for Wdfy3 in the establishment of forebrain long-range connectivity^18^. This study aligns with our own observations that Wdfy3 is required for *de novo* generation of axons and axon guidance (as evidenced by the pathway analyses of our proteomic survey), as well as for the correct localization of glial guidepost cells as shown in the *Wdfy3*^*disc*^ mice^16^.

Defective mitochondrial quality control provides a link to synaptic plasticity, as proper synaptic function strongly depends on effective mitochondrial turnover to maintain local bioenergetic requirements. In support of this notion, several proteins involved in mitophagy (e.g., Parkin, Pink1, Optn, Sqstm1/p62, Tbk1, and Ndp52, among others^53^) have been implicated in neurodegeneration. Furthermore, the majority of human cases reported to date carrying gain or loss of *Wdfy3* copy number variation (9 of 16) are affected by intellectual disability, global developmental delay and ASD (Supplementary Fig. S5), while other cases carrying either *de novo* nonsense or frameshift alleles, shown under Fig. 1A, have also been associated with ASD and intellectual disability^8,10–12^. The connection between Wdfy3 and behavior in our model of Wdfy3 haploinsufficiency is manifested as a significantly reduced rearing success of *Wdfy3*^+/*lacZ*^ dams, likely the result of reduced attentiveness towards their pups. In support of this premise, pups raised by WT dams survived at greater rates irrespective of pup genotype. But beyond survival, it is important to highlight that maternal care modulates neurobiological systems that impact long-term cognitive, social, and emotional development of the offspring^54–56^. Increased perinatal mortality was also reported in different *Wdfy3* mouse models, but was attributed to a selective maternal rejection of *Wdfy3* mutant pups due to deficits in their righting reflex^18^. However, while our haploinsufficient pups did not show any major developmental issues (none of 16 developmental milestones including righting reflex from PND2-PND14 reached statistical significance), the fostering experiments do not support this mechanism. Instead, Eph-ephrin signaling, one of the pathways identified as affected by Wdfy3 deficiency (Supplementary Dataset, under “Pathway analysis-reactome” tab), appears of particular interest. Our proteomic data showed a significant decrease in cortical EphA4 (log2 FC = −0.65; Fig. 9). EphA4 is implicated in the development of the corticospinal tract^57^, axon guidance for specific subsets of motor neurons^58,59^, neuroblast and glia organization^60^ and cerebellar wiring^61^. Its homozygous deletion results in impaired short-term habituation and novelty detection^62^. To date, no data are available in the literature on the role of EphA4 on nurturing behavior; however, the loss of the paralog EphA5, which has comparable embryonic and adult expression in brain, showed, in agreement with our study, a decreased survival rate in pups that were born and reared by *EphA5*^−/−^ females compared to those born and reared by WT (55% vs. 80%) and the higher mortality was directly correlated to altered maternal rearing behavior^63^. Thus, it is reasonable to assume that adaptation to the nurturing response requires substantial neuronal circuit remodeling, which will likely increase energy demand as well as autophagic flux, a concept supported by the fact that defective autophagy impairs synaptic pruning and neuronal remodeling, both processes also reportedly affected in ASD^28,64,65^. In other models, the link between Wdfy3 and atypical behaviors, including ASD, has been previously reported as a loss of habituation (suppression of startle response to a repeated non-adverse stimulus) in *Drosophila* subjected to inducible Wdfy3 knockdown in neurons^8^. Notably, habituation is a prerequisite for higher cognitive functions^66^, and altered habituation may substantially contribute to defective filtering, also identified as one of the features in ASD^67^.

Overall, our findings support the concept that Wdfy3 is critical for the maintenance of mitochondria quality control by regulating their turnover through mitophagy, a process required for sustaining neuronal circuitries. Deficits in recycling of building blocks, a lower capacity to clear damaged, unfolded or no-longer required proteins, along with the accumulation of dysfunctional mitochondria all may affect synaptic pruning as well as remodeling of neural circuits. The occurrence of these processes may all set the stage for energy shortage and neurotransmission imbalances, especially critical at the projections where mitochondria are essential for synaptic plasticity and neurotransmitter metabolism.

## Methods

### Animal breeding and husbandry

*Wdfy3*^*lacZ*^ (*Wdfy3*^*tm1a(KOMP)Mbp*^) mice were generated and genotyped as previously described^16^ and maintained on C57BL/6NJ background as a mixed WT/heterozygous mutant colony in facilities approved by the Association for Assessment and Accreditation of Laboratory Animal Care International (AALAC). All mice were genotyped for *Nnt* as described before^68^ (Supplementary Fig. S3). The animals were housed in Plexiglas cages (2–4 animals per cage; 55 × 33 × 19) and maintained under standard laboratory conditions (21 ± 2 °C; 55 ± 5% humidity) on a 12 h light/dark cycle, with *ad libitum* access to both water and food. The mice were fed with a standard rodent chow. All animals were handled in accordance with protocols approved by the University of California at Davis Institutional Animal Care and Use Committee. All protocols using animals followed the “Principles of laboratory animal care” (NIH publication No. 86-23, revised 1985).

### Mitochondrial outcomes

Mice (3–4 m old) were utilized for testing mitochondrial function in the indicated brain regions. After euthanasia, brains were dissected out of the skull and the cerebral hemispheres and cerebellum of each animal collected separately in ice-cold MSHE buffer (0.21 M mannitol, 0.07 M sucrose, 1 mM EDTA, 1 mM EGTA, 10 mM HEPES, pH 7.4). Subsequently, mitochondria were isolated by differential centrifugation as described before^68^. The mitochondrial fractions were heavily enriched with mitochondrial proteins presenting minimal contamination with cytosolic components (Fig. 8A,B). Mitochondria isolated from the indicated brain regions were evaluated by polarography under phosphorylating and non-phosphorylating conditions with NAD- and FAD-linked substrates^68^. Briefly, oxygen consumption was evaluated using a Clark-type oxygen electrode (Hansatech, King’s Lynn, UK) as described^4^. Mitochondria (10–20 µg protein) were added to the oxygen chamber in a buffer containing 0.22 M sucrose, 50 mM KCl, 1 mM EDTA, 10 mM KH_2_PO_4_, and 10 mM HEPES, pH 7.4. ATP-driven oxygen consumption rates were evaluated in the presence of (i) 1 mM ADP plus 1 mM malate-10 mM glutamate followed by the addition of 5 μM rotenone; (ii) 10 mM succinate followed by the addition of 3.6 μM antimycin A. The activities of mitochondrial NAD-derived ATP production and FAD-derived ATP production were evaluated as the difference of oxygen uptake recorded before and after the addition of rotenone and antimycin A, respectively. Specific activity of Complex IV was measured by polarography as described before^4^. Citrate synthase activity was measured spectrophotometrically as described before^68^. ROS/proton leak was calculated as the ratio of the succinate-supported oxygen uptake resistant to 2 µM oligomycin and the basal oxygen consumption. SRC (spare respiratory capacity) was expressed as the ratio between maximum oxygen consumption in the presence of 10 mM succinate and 20 nM of the uncoupler FCCP and the basal one.

### Proteomics of cortical mitochondria-enriched and MDVs fractions

*Sample Preparation –*Cortices from 3-m old 7 WT and 7 female haploinsufficient mice were used to obtain mitochondria-enriched fractions as described before^68^ and MDV-enriched fractions essentially as described by others^35^. Protein evaluation was performed with Pierce BCA protein assay (Thermo Scientific, Waltham MA). *Mass Spectrometry -* Samples were submitted to the UC Davis Genome Center Core Proteomics Facility for liquid chromatography with tandem mass spectrometry. Protein pellets were digested overnight with a trypsin to protein ratio of 1:30. The equivalent of 2–5 µg of protein was loaded into the LC-MS/MS. *Database Searching -* All MS/MS samples were analyzed using X! Tandem (The GPM, thegpm.org; version TORNADO (2010.01.01.4)). X! Tandem was set up to search the uniprot__20120523_gTmkm3 database (89576 entries) assuming the digestion by the enzyme trypsin. X! Tandem was searched with a fragment ion mass tolerance of 20 ppm and a parent ion tolerance of 1.8 Da. Deamidation of Asn and Gln, oxidation of Met and Trp, sulphone of Met, Trp oxidation to formylkynurenin, and acetylation of the *N*-terminus were specified in X! Tandem as variable modifications. *Criteria for Protein Identification -* Scaffold (v. 3.00.07, Proteome Software Inc., Portland, OR) was used to validate MS/MS based peptide and protein identification. Peptide identification was accepted if it could be established at >80% probability by the Peptide Prophet algorithm^69^. Protein identifications were accepted if they could be established at >80.0% probability and contained at least 2 identified peptides. Protein probabilities were assigned by the Protein Prophet algorithm^70^. Proteins that contained similar peptides and could not be differentiated based on MS/MS analysis alone were grouped to satisfy the principles of parsimony. *Proteomics analysis*-The proteome profiles from all 14 animals were normalized by their spectral counting sum and analyzed by using PLS-DA to identify the features that separated the most the two genotypes. The proteins that best described the differences between the two groups were selected by setting a variable in importance projection (VIP) score of >0.8 (Fig. 5). From these, those associated with the gene ontology term “autophagy” or “mitophagy” (under “biological process”) or “mitoch*” (under “cellular compartment”) and with a *p*-value corrected by the FDR of 0.05 or lower, were selected. From this subset, only those with a |LOG2 ratio| ≥ 0.5 were kept (Fig. 9**)**.

To compute the achieved power for the analysis of the abundance of mitochondrial proteins in the cortex MDV fraction in WT and haploinsufficient mice, a post-hoc analysis to a two-tailed Student’s t test (calculated with G*Power, version 3.1.9.2) was run, given an alpha of 0.05 and a sample size of 109 proteins. The results indicated that the achieved power was >99%.

### Western blotting

Mitochondria-enriched and cytosolic fractions were isolated from cortex, hippocampus, cerebellum and olfactory bulb of WT and *Wdfy3*^+/*lacZ*^ mice as described before^68^. Thirty-five µg of proteins were solubilized in SDS sample buffer (Life Technologies, Grand Island, NY) and loaded onto a 4–12% bis-tris gel (Life Technologies) as previously described^68^. After transferring proteins with an iBlot apparatus (Life Technologies), membranes were blocked with LI-COR blocking buffer (LI-COR Biosciences, Lincoln, NE) for 1 h at 20 °C and subsequently probed overnight at 4 °C with the following antibodies: anti-Lamp2 (Abcam, Cambridge, MA; 1:1,000 dilution), anti-LC3 (Novus Biologicals, Littleton, CO; 1:1,000 dilution), anti-Mfn2 (Proteintech, Rosemont, IL; 1:500 dilution), anti-MnSOD (Millipore, Billerica, MA; 1:1,000 dilution), anti Sqstm1 (Cell Signaling Technology, Danvers, MA; 1:500 dilution), anti-Park2 (Abcam; 1:500 dilution), and anti-Pink1 (Novus Biologicals; 1:1,000 dilution). As a loading control, we used anti-β-actin antibody (Sigma, St. Louis, MO; 1:20,000 dilution, 1 h at 20 °C). Secondary antibodies were from LI-COR (Lincoln, NE; 1:10,000 dilution). Membranes were visualized with the use of the Odyssey Infrared Imaging System (LI-COR) and densitometry analysis carried out with ether the Carestream or ImageJ softwares.

### Cell culture of neuronal progenitor cells (NPC)

Murine striatal neuronal progenitor cells (NPCs) were described in detail and characterized elsewhere^71^. Cells were grown in high glucose DMEM (Thermo Fisher Scientific, Waltham, MA) supplemented with 10% FBS (Hyclone, Logan, UT), 100 units/ml penicillin, 100 µg Streptomycin/ml and 0.4 mg/ml G418 (Thermo Fisher Scientific, Waltham, MA) at 33 °C in 5% CO_2_, never exceeding passage #10. For silencing experiments, siRNA-lipid complexes were formed using 1 ml Opti-MEM I Medium (Gibco) without serum, 43 μl of Lipofectamine RNAiMAX (Thermo Fisher Scientific) and 145 pmol scrambled siRNA or *Wdfy3* siRNA (Thermo Fisher Scientific, #AM4611 and AM16708, ID: 95113, respectively). Cells were then added to the siRNA-lipid complex at a concentration of 1 × 10^6^ in 9 ml of growth medium. After 48 h, cells were collected and viability (>85%) checked by trypan blue staining (Sigma-Aldrich, St. Louis, MO) with the use of a TC20 Automated Cell Counter (Bio-Rad, Hercules, CA). Total RNA was extracted from cells using the RNAeasy mini kit (Qiagen, Hilden, Germany) according to the manufacturer’s protocol. Concentration and purity of RNA was measured at 260 nm and 280 nm on a Tecan Infinite M200 Nanoquant (Tecan, Austria). Subsequently, QuantiTect Reverse Transcription Kit (Qiagen) with provided RT Primer Mix was used for cDNA synthesis. Assessment of *Wdfy3* silencing efficiency was performed by qPCR using iQ SYBR Green Supermix (Bio-Rad) according to the manufacturer’s protocol. *Wdfy3* gene expression levels were assessed using forward 5′CTGAATGGGGCCAGATCCTC-3′ and reverse primer 5′-AAGGAGAGCCTGCTTGAGTG-3′. For gene expression fold change analysis, the delta-delta Ct method was used. Gene expression levels were normalized to *Gapdh* expression used as a housekeeping gene.

### Protocol and analyses of immunofluorescence by confocal microscopy

Cells (either primary cortical neurons from mice or NPCs, 1 × 10^5^) were seeded on sterile cover slips and grown over night at 37 °C. After desired confluence was achieved, cells attached to coverslips were stained with 500 nM of MitoTracker Deep Red FM (Molecular Probes Inc., Eugene, OR, USA) diluted in growth media, as previously described^71^, fixed in 3.7% formaldehyde for 10 min, and subsequently blocked/permeabilized for 30 min in 20% goat serum, 0.1% BSA, 0.2% Triton X-100 in phosphate-buffered saline (GS-PBS)^71^. Evaluation of total mitochondrial mass and distribution was obtained in NPCs by staining with anti-ATP5B antibody (1:200 dilution in GS-PBS; BD Biosciences, San Jose, CA), overnight at 4 °C. For evaluation of LC3 or Wdfy3 levels, upon fixation and blocking/permeabilization of the cells, NPCs were probed with anti-LC3 antibody (Cell Signaling, Danvers, MA) and anti-Wdfy3 antibody (Abcam, Cambridge, MA), both at 1:100 dilution in GS-PBS overnight at 4 °C. The following day, cells were washed in PBS and probed with a goat anti-rabbit or goat anti-mouse AlexaFluor 488 antibody (1:1.000 in 2% GS-PBS; LI-COR, Lincoln, NE) for 1 h at 20 °C. NPCs were subsequently counterstained with 1 µg/ml 4′,6 diamidino-2-phenylindole (DAPI) and mounted on glass slides with ProLong Gold anti-fading mounting media (Thermo Fisher Scientific, Waltham, MA). Fluorescent images were obtained using a Leica TCS SP8 *STED 3*X confocal microscope (Leica, Buffalo Grove, IL) and analyzed with the Leica LAS X Core software. De-convolution was carried out with the use of the Huygens Professional software. Self-crossing in cortical neuronal projections was visualized with ImageJ and their frequencies counted manually. The projections were identified by using an algorithm within ImageJ to recognize soma vs. projections. For mitochondrial morphology quantification, images were further analyzed with two macro tools designed for ImageJ (Fiji)^72,73^ to allow the quantification of mitochondrial mass, morphological features and network integrity.

### Brain whole-mount imaging

Animals were euthanized, and tissues fixed by transcardial perfusion with 4% PFA (paraformaldehyde) in PBS. After being removed from the skulls, brains were submerged in PBS and imaged using a Zeiss Lumar.V12 stereo-microscope (Oberkochen, Germany) with attached Zeiss AxioCam MRc camera and associated AxioVision software (version 4.8.2).

### Hematoxylin-eosin (HE) staining

Animals were anesthetized, and tissues fixed by transcardial perfusion with 4% PFA in PBS. Brains were dissected, dehydrated through a series of graded ethanol baths, and then infiltrated with paraffin at 65 °C. The infiltrated tissues were embedded into wax blocks, cut to 10 µm sections that were mounted on glass slides, and processed for HE-staining according to standard methods. Photomicrographs were acquired on an Olympus BX61 microscope with Olympus DP71 camera and associated Olympus cell Sens Dimension software at 4X magnification.

### X-gal staining

Staining for β-galactosidase (β-gal) activity expressed by the *lacZ* reporter transgene inserted into the targeted *Wdfy3* locus was performed by using X-gal (5-bromo-4-chloro-3-indolyl-β-D-galactopyranoside) as a substrate according to standard protocols as previously described^74^. In brief, animals were anesthetized, and tissues mildly fixed by transcardial perfusion with 2% PFA in PBS. Brains were dissected, processed through 15% and 30% sucrose/PBS and cryoprotectively frozen in OCT compound (Sakura Finetek USA Inc., Torrance, CA). Subsequently, sections were cut at 40 μm, mounted on glass slides, stained, and counterstained with nuclear fast red (Sigma). Photomicrographs were acquired on an Olympus BX61 microscope with Olympus DP71 camera and associated Olympus cellSens Dimension software at 4X magnification.

### Statistics

Unless otherwise stated, statistical analysis was performed with two-tailed Mann-Whitney test or by Kruskal-Wallis followed by Dunn’s multiple comparisons test. All significance levels were set at *p* ≤ 0.05.

## Electronic supplementary material

Supplementary Material

Supplementary Dataset 1
