# Supplementary material for: Beyond autophagy: a novel role for autism-linked Wdfy3 in brain mitophagy
Source: Sci Rep. 2018 Jul 27;8:11348. doi: 10.1038/s41598-018-29421-7 (PMC6063930; doi:10.1038/s41598-018-29421-7)
Supplement: Supplementary file 1 — Supplementary Material [file 41598_2018_29421_MOESM1_ESM.pdf]

Supplementary Material

Beyond autophagy: novel role for the autism-linked Wdfy3 gene in brain mitophagy

Eleonora Napoli[1], Gyu Song[1], Alexios Panoutsopoulos[2,3], M. Asrafuzzaman Riyadh[2,3], Gaurav Kaushik[2,3], Julian Halmai[1], Richard Levenson[2,4,5], Konstantinos Zarbalis[2,3*], and Cecilia Giulivi[1,5*]

[1] Department of Molecular Biosciences, School of Veterinary Medicine, University of California, Davis, CA 95616;

[2] Department of Pathology and Laboratory Medicine, University of California Davis;

[3] Institute for Pediatric Regenerative Medicine, Shriners Hospitals for Children, Northern California, 2425 Stockton Boulevard, Sacramento, CA 95817;

[4] Department of Psychiatry and Behavioral Sciences, University of California Davis School of Medicine, Sacramento, CA, 95817;

[5] Medical Investigations of Neurodevelopmental Disorders (MIND) Institute, University of California Davis, CA 95817

*Corresponding authors: Dr. Cecilia Giulivi: cgiulivi@ucdavis.edu; Dr. Konstantinos Zarbalis: kzarbalis@ucdavis.edu.

Glossary of terms

Aggrephagy-(GO:0035973) Selective degradation of protein aggregates by macroautophagy. Source: GOC:autophagy, PMID:18508269, GOC:kmv, PMID:25062811.

Axonogenesis-(GO:0007409) *De novo* generation of a long process of a neuron, including the terminal branched region. Refers to the morphogenesis or creation of shape or form of the developing axon, which carries efferent (outgoing) action potentials from the cell body towards target cells. Source: GOC:pg, GOC:dph, GOC:jid, GOC:pr, ISBN:0198506732.

Bioenergetics-It is the quantitative study of the energy transductions that occur in living cells and on the nature and function of the chemical processes underlying these transductions[1]. In our study, it is implied that we were focusing on mitochondrial bioenergetics.

Macroautophagy-(GO:0016236) The major inducible pathway for the general turnover of cytoplasmic constituents in eukaryotic cells, it is also responsible for the degradation of active cytoplasmic enzymes and organelles during nutrient starvation. Macroautophagy involves the formation of double-membrane-bounded autophagosomes which enclose the cytoplasmic constituent targeted for degradation in a membrane-bounded structure. Autophagosomes then fuse with a lysosome (or vacuole) releasing single-membrane-bounded autophagic bodies that are then degraded within the lysosome (or vacuole). Some types of macroautophagy, e.g. pexophagy, mitophagy, involve selective targeting of the targets to be degraded. Source: PMID:9412464, PMID:12914914, PMID:11099404, PMID:20159618, PMID:16973210, PMID:15798367.

Micromitophagy-(GO:0000424) Degradation of a mitochondrion by lysosomal microautophagy. Source: PMID:27003723, PMID:15798367. Mitochondria-derived vesicle formation (micromitophagy or Type 3 mitophagy). Includes the removal of damaged mitochondria components by forming mitochondria-derived vesicles (MDV) that bud off and then transit to lysosomes[2].

Mitochondrial dynamics-as utilized in this study refers to the spatial mitochondria positioning within cell (GO:0048312; GO:0048311), mitochondrial shape changes (GO:0070584) and assembly, arrangement of constituent parts, or disassembly of a mitochondrion including distribution (GO:0007005).

Mitophagy-(GO:0000423) Degradation of a mitochondrion by macroautophagy. Source: PMID:15798367. Clearance of damaged mitochondria via autophagy of mitochondria.

Oxidative phosphorylation (OXPHOS)-(GO:0006119) The phosphorylation of ADP to ATP that accompanies the oxidation of a metabolite through the operation of the respiratory chain. Oxidation of compounds establishes a proton gradient across the membrane, providing the energy for ATP synthesis. Source:ISBN:0198506732, ISBN:0471331309.

Note: All definitions were obtained from [3,4], unless noted otherwise.

Supplementary Tables

Table S1 Mitochondrial outcomes in cortex from WT and Wdfy3$^{+/lacz}$ mice

Outcome	Mean ± SEM		
	WT (n = 11)	Wdfy3$^{+/lacZ}$ (n = 16)	*p*-value
ATP-driven oxygen uptake			
Substrate			
Malate-glutamate	30 ± 2	23 ± 2	**0.012**
Succinate	55 ± 3	40 ± 2	**0.0001**
Markers of mitochondrial mass and compartments			
Complex IV activity (MIM)	77 ± 3	52 ± 4	**<0.0001**
Citrate synthase activity (MM)	450 ± 16	492 ± 17	**0.049**
CCO/CS (MIM/MM)	0.17 ± 0.01	0.11 ± 0.02	**0.014**
Others			
SRC	2.48 ± 0.49	0.96 ± 0.03	**0.0005**
ROS/Proton leak	0.43 ± 0.03	0.76 ± 0.05	**< 0.0001**

Markers of mitochondrial mass and compartments: Complex IV or Cytochrome *c* oxidase activity (CCO), as a marker of mitochondrial inner membrane (MIM), was evaluated by polarography as described in the Methods section. Citrate synthase activity, as a marker of mitochondrial matrix (MM), was expressed as nmol x (min mg protein)$^{-1}$. SRC (spare respiratory capacity) is expressed as the difference between maximum oxygen consumption (in the presence of the uncoupler FCCP) and the basal one, normalized by basal respiration. ROS/proton leak is calculated as the ratio between oligomycin-resistant and basal oxygen consumption. All *p*-values were calculated using a 1-tailed *t*-test. In bold, values with $p \leq 0.050$.

Table S2 Mitochondrial outcomes in cerebellum from WT and Wdfy3$^{+/lacz}$ mice

Outcome	Mean ± SEM		
	WT (n = 11)	Wdfy3$^{+/lacZ}$ (n = 16)	*p*-value
ATP-driven oxygen uptake			
Substrate			
Malate-glutamate	43 ± 5	33 ± 3	**0.040**
Succinate	92 ± 10	71 ± 5	**0.025**
Markers of mitochondrial mass and compartments			
Complex IV activity (MIM)	128 ± 8	97 ± 8	**0.007**
Citrate synthase activity (MM)	538 ± 22	493 ± 15	**0.046**
CCO/CS (MIM/MM)	0.24 ± 0.02	0.19 ± 0.01	**0.011**
Others			
SRC	1.60 ± 0.03	1.42 ± 0.04	**0.001**
ROS/Proton leak	0.45 ± 0.003	0.59 ± 0.02	**< 0.0001**

All details were indicated under Table S1 legend. All *p*-values calculated using the 1-tailed *t*-test. In bold, values with $p \leq 0.050$.

Table S3 Mitochondrial outcomes in hippocampus from WT and Wdfy3$^{+/lacz}$ mice

**Outcome**	**Mean ± SEM**		
	WT (n = 3)	Wdfy3$^{+/lacZ}$ (n = 5)	*p*-value
ATP-driven oxygen uptake			
Substrate			
Malate-glutamate	29 ± 4	31 ± 5	0.396
Succinate	11 ± 3	15 ± 5	0.295
Markers of mitochondrial mass and compartments			
Complex IV activity (MIM)	70 ± 3	61 ± 6	0.160
Citrate synthase activity (MM)	99 ± 8	124 ± 9	0.057
CCO/CS (MIM/MM)	0.18 ± 0.01	0.12 ± 0.01	**0.004**
Others			
SRC	1.09 ± 0.12	1.29 ± 0.68	0.417
ROS/Proton leak	0.67 ± 0.05	0.76 ± 0.17	0.354

All details were indicated under Table S1 legend. All *p*-values calculated using the 1-tailed *t*-test. In bold, values with $p \leq 0.050$.

Table S4 Mitochondrial outcomes in olfactory bulb from WT and Wdfy3$^{+/lacz}$ mice

**Outcome**	**Mean ± SEM**		
	WT (n = 3)	Wdfy3$^{+/lacZ}$ (n = 5)	*p*-value
ATP-driven oxygen uptake			
Substrate			
Malate-glutamate	25.0 ± 0.3	28 ± 4	0.297
Succinate	17.3 ± 0.9	19 ± 3	0.345
Markers of mitochondrial mass and compartments			
Complex IV activity (MIM)	86 ± 8	100 ± 11	0.205
Citrate synthase activity (MM)	141 ± 1	156 ± 8	0.105
CCO/CS (MIM/MM)	0.15 ± 0.01	0.16 ± 0.01	0.268
Others			
SRC	0.72 ± 0.08	0.58 ± 0.36	0.391
ROS/Proton leak	0.75 ± 0.05	0.68 ± 0.21	0.406

All details were indicated under Table S1 legend. All *p*-values calculated using the 1-tailed *t*-test.

Supplementary Figures and Figure Legends

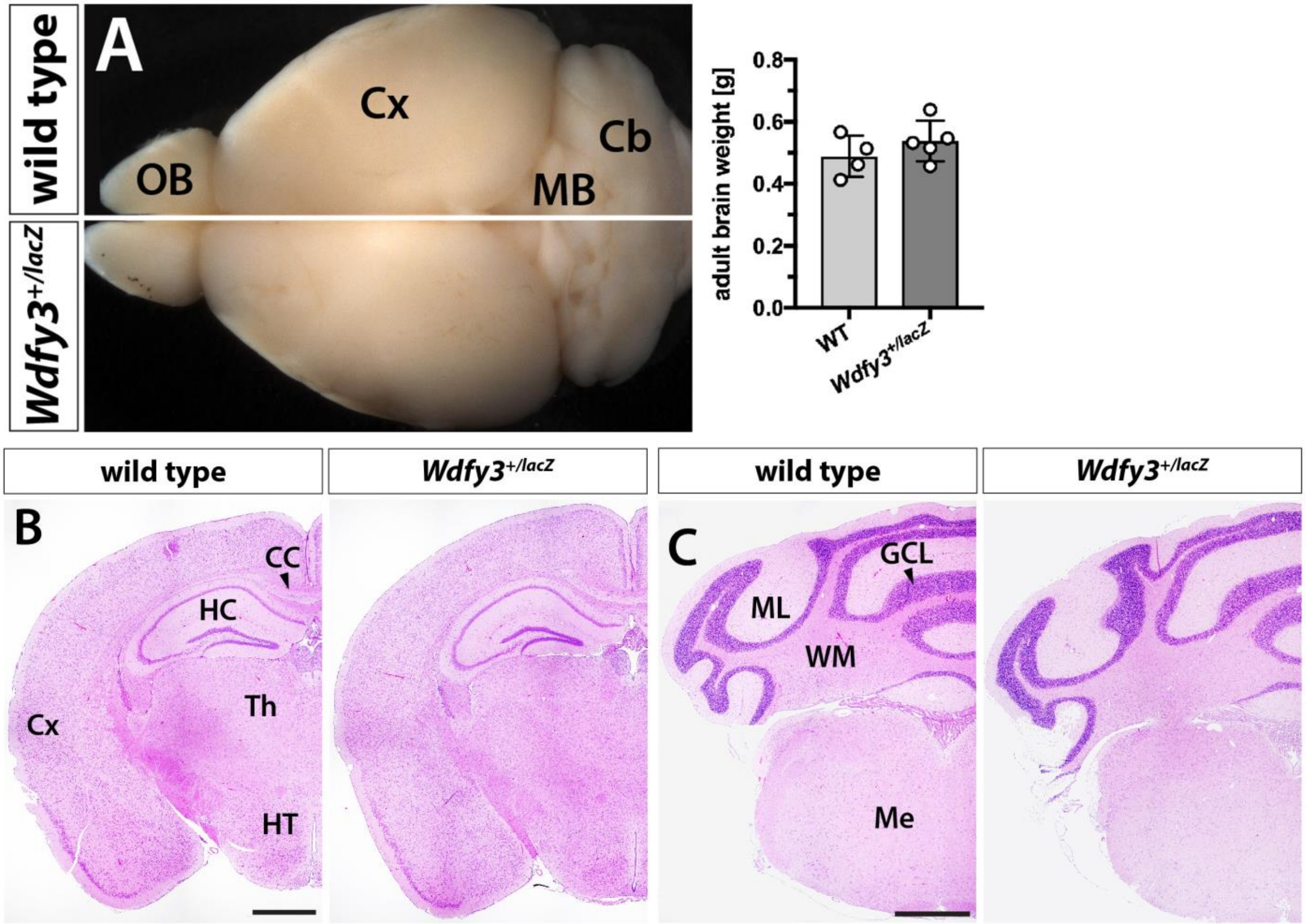

Supplementary Figure S1 Brain morphology of *Wdfy3*$^{+/lacZ}$ mice

A. Left panel. Top views of 3-m old whole-mount brains illustrate the absence of overt morphological anomalies in ***Wdfy3***$^{+/lacZ}$ mutants. **Right panel.** Analysis of brain wet weight confirmed no significant differences between genotypes (Student's *t* test, *p*=0.301). **B-C.** Paraformaldeyhyde-perfused brains were sectioned, routinely processed for histology, embedded in paraffin, sectioned for slide mounting at 3-5 µm and stained with hematoxylin and eosin. Coronal HE-stained forebrain (**B**) and cerebellum sections (**C**) of 3-m old (PND100) *Wdfy3*$^{+/lacZ}$ and WT brain reveal comparable morphology with no overt abnormalities. CC, corpus callosum; Cx, Cortex; GCL, granular cell layer; HC, hippocampus; HT, hypothalamus; MB, midbrain; Me, medulla; ML, molecular layer; Th, thalamus; WM, white matter. Scale bars are 1 mm.

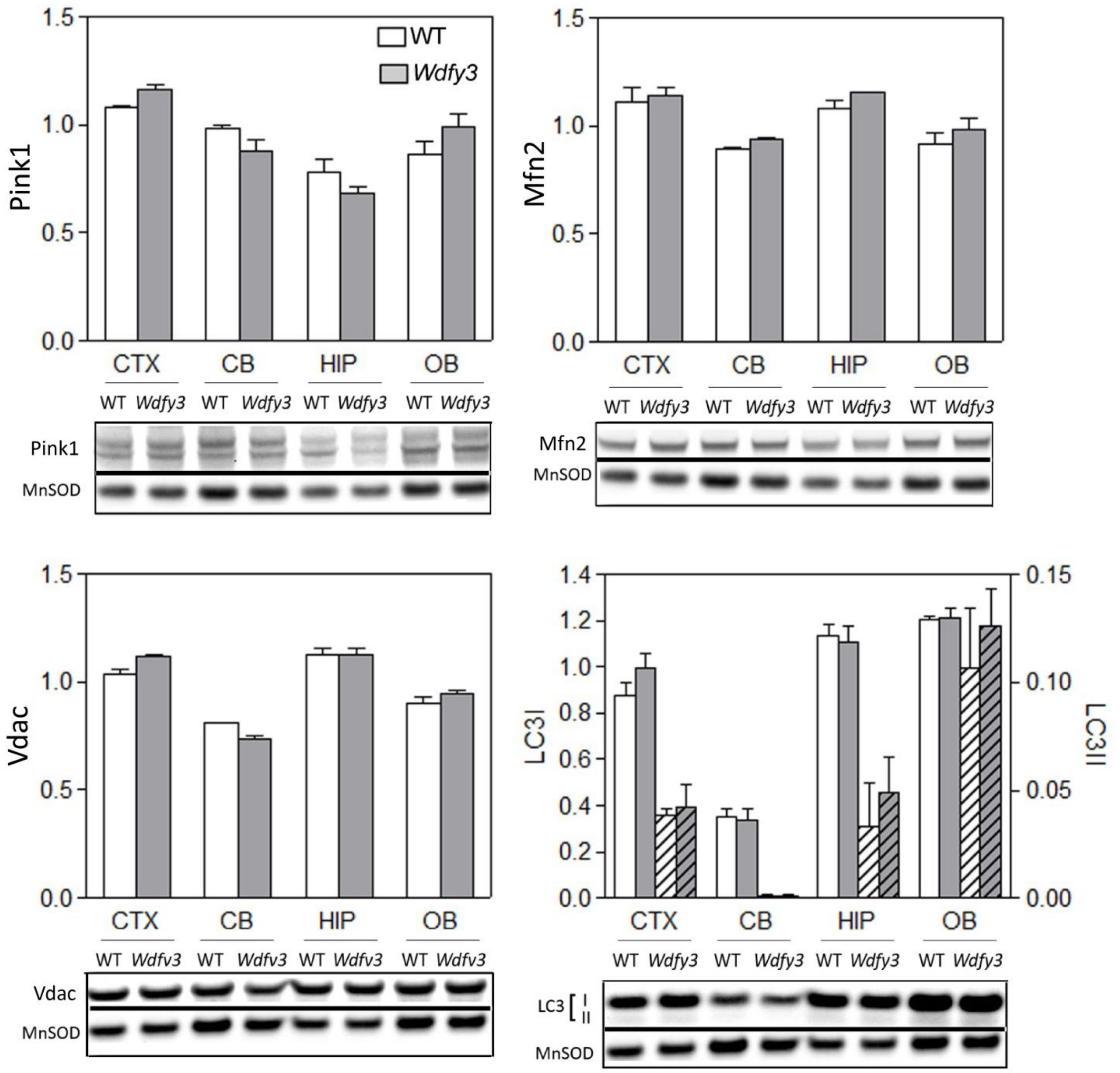

Supplementary Figure S2 Expression levels of markers of mitophagy and autophagy
Representative Western blots and densitometry of mitophagy-related proteins (Pink1, Mfn2, Vdac and LC3I and LC3II) in mitochondrial-enriched fractions obtained from cortex (CTX), cerebellum (CB), hippocampus (HIP), and olfactory bulb (OB) of WT (white bars) and W*dfy3* (gray bars) haploinsufficient mice. Data are shown as mean ± SEM of samples ran in duplicates (*n* = 3 WT and 5 mutants). Statistical analysis was performed with Student's *t* test between WT and W*dfy3* mice for each brain area. MnSOD was used as mitochondrial loading control.

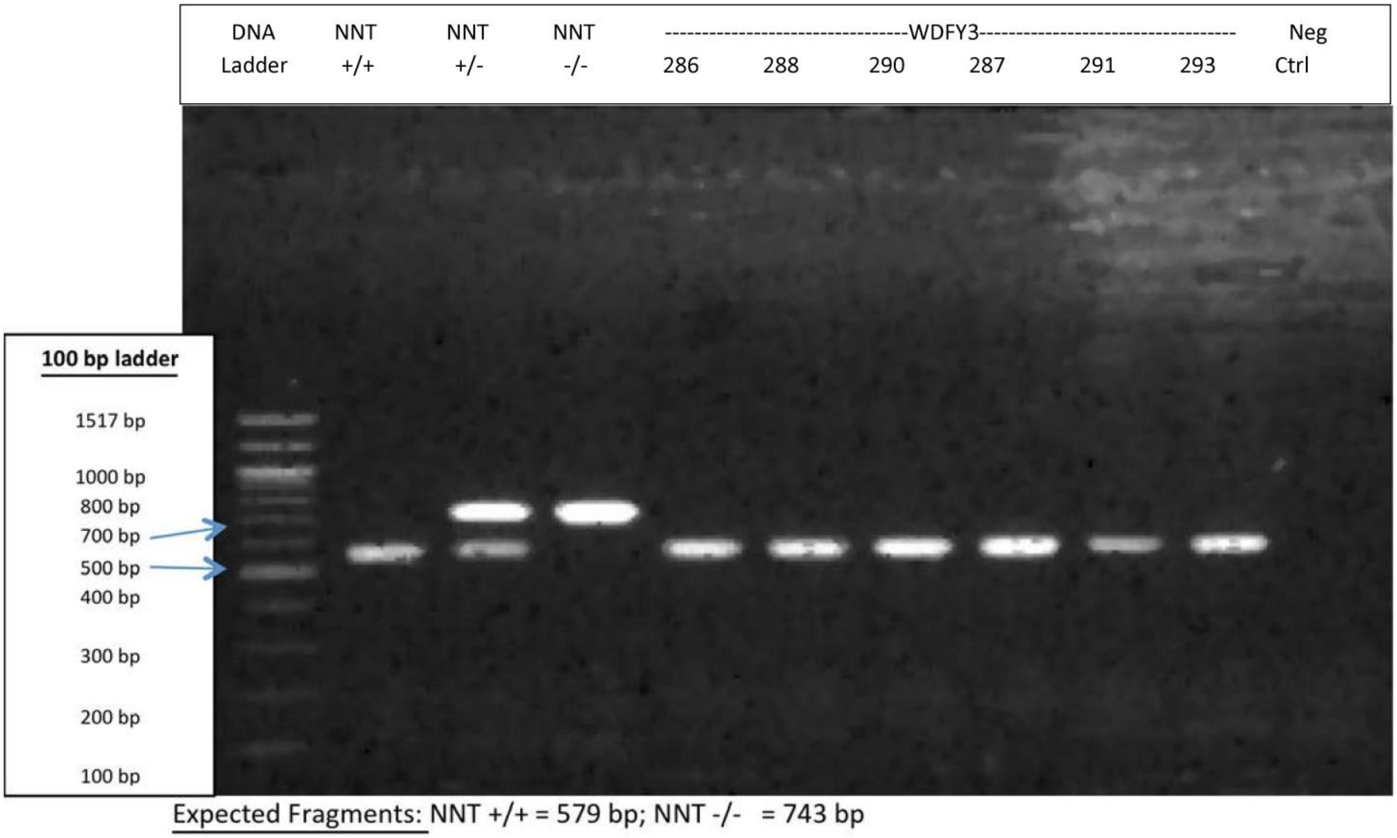

Supplementary Figure S3 Representative *Nnt* genotyping

All mice utilized in this study were genotyped for the wild-type or truncated *Nnt* gene as described [5]. Genomic DNA was isolated from 5 mg of hindbrain as previously described [6]. Concentration and purity of DNA was evaluated by measuring the absorbance of 260 and 280 nm on a Tecan Infinite M200 Nanoquant (Tecan, Austria). The criteria utilized for the presence of *Nnt* was the occurrence of a band at the expected size of 579 bp; the truncated form shows a band of an expected size of 743 bp; heterozygous carriers showed two bands, each at each expected size. Labels: NNT+/+, +/- and -/- are positive controls; the numbers indicate the mouse numbers utilized in this study; Neg Ctrl= negative control (no DNA added).

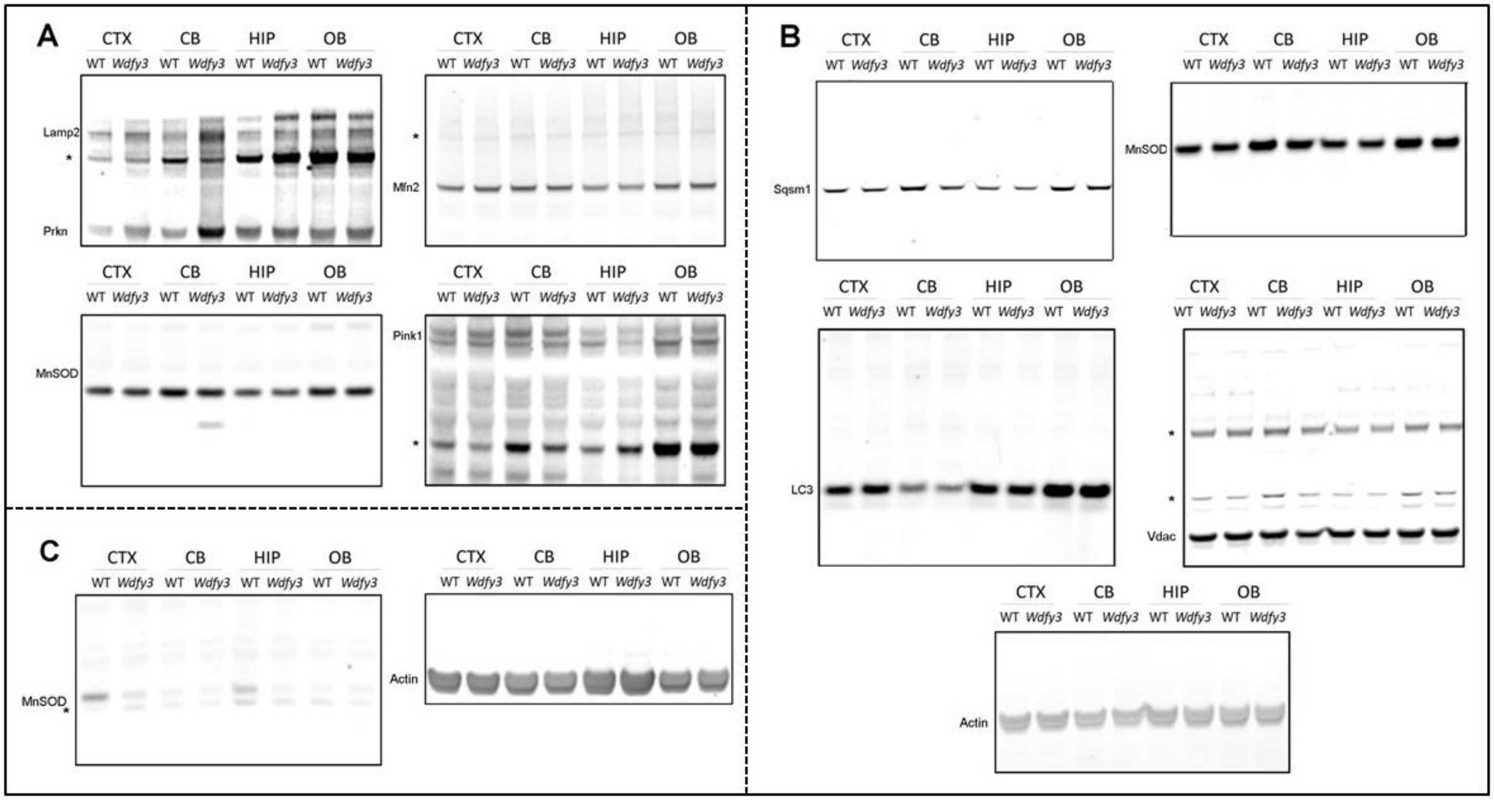

Supplementary Figure S4 Full-length western blots images
Representative full-length, black and white images of Western blots shown as cropped images under **Fig. 8** and **Supplementary Fig. 1**. In many instances, the scan of the membrane was performed in the area proximal to the expected molecular weight of the protein of interest and, as such, a full version of the image was not acquired. Membrane shown in **A** was probed for Prkn, Mfn2, Lamp2, Pink1 and MnSOD. Membrane shown in **B** was probed for Sqsm1, LC3, MnSOD, and VDAC. In **C** are shown cytosolic levels of MnSOD and actin (see **Fig. 8A-B**). Images were acquired with the use of the Odyssey Infrared Imaging System (LI-COR Biosciences), which allows the sequential detection of multiple proteins when probed with antibodies raised in different species (i.e., rabbit and mouse), due to the possibility of detecting IR-labeled secondary antibodies simultaneously in both 700 and 800 nm channels in a single scan. Asterisks indicate unspecific bands.

DECIPHER ID	Variant	Sex	Size	Pathogenicity Contribution ?	Inheritance	Phenotype(s)	Patient Open-Access Variants
994	4 81789569 92857255 deletion	46XX	11.07 Mb	Unknown	De novo constitutive	Broad palm, Intellectual disability, Narrow nasal bridge, Short foot, Short palm, Short philtrum, Short stature, Tapered finger, Thin lower lip vermilion, Thin upper lip vermilion	2
994	4 82210925 91215314 deletion	46XX	9.00 Mb	Unknown	De novo constitutive	Broad palm, Intellectual disability, Narrow nasal bridge, Short foot, Short palm, Short philtrum, Short stature, Tapered finger, Thin lower lip vermilion, Thin upper lip vermilion	2
1599	4 71660502 89588610 loss	46XX	17.93 Mb	Unknown	Unknown		1
4665	4 82010051 86491895 deletion	46XX	4.48 Mb	Unknown	De novo constitutive	Aplasia/Hypoplasia of metatarsal bones, Clinodactyly of the 5th finger, Delayed speech and language development, Intellectual disability, Short palm, Short stature, Short toe	1
249573	4 84946652 88878077 deletion	unknown	3.93 Mb	Unknown	Unknown	Abnormality of the skin, Atria septal defect, Behavioral abnormality, Blepharophimosis, Epicanthus, Intellectual disability	1
254502	4 73192275 87339183 loss	46XX	14.15 Mb	Unknown	De novo constitutive		1
256563	4 74201207 110610111 duplication	46XX	36.41 Mb	Unknown	Imbalance arising from a balanced parental rearrangement	Absent speech, Coloboma, Intellectual disability, profound, Moderate postnatal growth retardation	1
261020	4 76264933 88265514 loss	46XX	12.00 Mb	Unknown	Unknown		1
261336	4 83155020 90292135 deletion	46XX	7.14 Mb	Unknown	De novo constitutive	Autism, Intellectual disability, Self-mutilation	3
272125	4 85145484 86152827 deletion	46XX	1.01 Mb	Unknown	Unknown	Abnormality of finger, Frontal bossing	1
272833	4 85777938 86923300 deletion	46XY	1.15 Mb	Unknown	Unknown	Intellectual disability	1
280470	4 76955357 86554329 deletion	46XY	9.60 Mb	Unknown	De novo constitutive	Global developmental delay	1
285906	4 82722961 86152968 deletion	46XX	3.43 Mb	Definitely pathogenic	Unknown	Absent gallbladder, Aplasia/Hypoplasia of the hallux, Bilateral cleft lip and palate, Hemiatrophy, Hypertelorism, Hypodysplasia of the corpus callosum, Hypoplastic sacral vertebrae, Intrauterine growth retardation, Laryngeal stenosis, Micrognathia, obsolete Malformation of the heart and great vessels, Short foot, Single umbilical artery, Unilateral narrow palpebral fissure	3
291279	4 83072226 85590900 deletion	46XX	2.52 Mb	Likely pathogenic	De novo constitutive	Neonatal hypotonia	1
292366	4 83008007 85642725 deletion	46XX	2.63 Mb	Likely pathogenic Full	De novo constitutive	Bicornuate uterus, Intellectual disability, severe	1
331528	4 85755841 86555973 gain	46XY	800.13 kb	Likely pathogenic	Maternally inherited, constitutive in mother	Autism, Behavioral abnormality	1
333579	4 85537264 100681416 deletion	46XX	15.14 Mb	Definitely pathogenic Full	De novo constitutive	Epicanthus, Global developmental delay, Low-set ears, Muscular hypotonia, Pectus excavatum, Short philtrum, Thin upper lip vermilion	1

Supplementary Figure S5 ***Wdfy3* genomic variants in humans and associated phenotypes**
Reported information was generated by the DECIPHER community.

References

1 Nelson, D. L., Cox, M. M. & Lehninger, A. L. *Lehninger principles of biochemistry*. 4th edn, (W.H. Freeman, 2005).

2 Lemasters, J. J. Variants of mitochondrial autophagy: Types 1 and 2 mitophagy and micromitophagy (Type 3). *Redox Biol*. **2**, 749-754, doi:10.1016/j.redox.2014.06.004 (2014).

3 The Gene Ontology, C. Expansion of the Gene Ontology knowledgebase and resources. *Nucleic Acids Res*. **45**, D331-D338, doi:10.1093/nar/gkw1108 (2017).

4 Ashburner, M. *et al.* Gene ontology: tool for the unification of biology. The Gene Ontology Consortium. *Nat Genet*. **25**, 25-29, doi:10.1038/75556 (2000).

5 Nicholson, A. *et al.* Diet-induced obesity in two C57BL/6 substrains with intact or mutant nicotinamide nucleotide transhydrogenase (Nnt) gene. *Obesity (Silver Spring)*. **18**, 1902-1905, doi:10.1038/oby.2009.477 (2010).

6 Giulivi, C. *et al.* Basal bioenergetic abnormalities in skeletal muscle from ryanodine receptor malignant hyperthermia-susceptible R163C knock-in mice. *J Biol Chem*. **286**, 99-113, doi:10.1074/jbc.M110.153247 (2011).
